# Dibutyl phthalate alters the metabolic pathways of microbes in black soils

**DOI:** 10.1038/s41598-018-21030-8

**Published:** 2018-02-08

**Authors:** Weihui Xu, Yimin You, Zhigang Wang, Wenjing Chen, Jin Zeng, Xiaosong Zhao, Yunpeng Su

**Affiliations:** 1 School of Life Science and Agriculture and Forestry, Qiqihar University, Qiqihar, Heilongjiang 161006 China; 2 Institute for Environmental Genomics, Department of Microbiology and Plant Biology, University of Oklahoma, Norman, OK 73072 USA; 3 State Key Laboratory of Lake Science and Environment, Nanjing Institute of Geography and Limnology, Chinese Academy of Sciences, Nanjing, 210000 China; 4 Key Laboratory of Urban Agriculture in South China, Ministry of Agriculture, Guangzhou, 510640 China

## Abstract

Dibutyl phthalate (DBP) is well known as a high-priority pollutant. This study explored the impacts of DBP on the metabolic pathways of microbes in black soils in the short term (20 days). The results showed that the microbial communities were changed in black soils with DBP. In nitrogen cycling, the abundances of the genes were elevated by DBP. DBP contamination facilitated 3′-phosphoadenosine-5′-phosphosulfate (PAPS) formation, and the gene flux of sulfate metabolism was increased. The total abundances of ABC transporters and the gene abundances of the monosaccharide-transporting ATPases *MalK* and *MsmK* were increased by DBP. The total abundance of two-component system (TCS) genes and the gene abundances of malate dehydrogenase, histidine kinase and citryl-CoA lyase were increased after DBP contamination. The total abundance of phosphotransferase system (PTS) genes and the gene abundances of phosphotransferase, *Crr* and *BglF* were raised by DBP. The increased gene abundances of ABC transporters, TCS and PTS could be the reasons for the acceleration of nitrogen, carbon and sulfate metabolism. The degrading-genes of DBP were increased markedly in soil exposed to DBP. In summary, DBP contamination altered the microbial community and enhanced the gene abundances of the carbon, nitrogen and sulfur metabolism in black soils in the short term.

## Introduction

The grain output from the black soil region accounts for 30% of the national staple food production in China^1^. Plastic films (for mulching cultivation) have been widely used in farm production of the region. Dibutyl phthalate (DBP) bound to the plastics relatively weakly via hydrogen bond or Van der Waals force, and not combine into PVC polymer chain^2^. It can be easily released into earth surface and groundwater, and is easy to accumulate^3^. DBP was detected in soils of all seasons, and the highest DBP concentration was determined in summer^4^. DBP is ubiquitous in soils^5^. The residual DBP levels reached 14.6 mg kg^−1^ in black soils and 29.37 mg kg^−1^ in fluvo-aquic soils^5^, and exceeded the recommended values in the soil cleanup guidelines (0.08 mg kg^−1^) adopted by the US Environmental Protection Agency (US EPA)^6,7^. DBP can lead to biological health problems, including developmental and reproductive toxicity^8^. DBP has been listed as one of priority pollutants by both the China National Environmental Monitoring Centre and the United States Environmental Protection Agency^9^ because of its mutagenicity, teratogenicity, and carcinogenicity^10^. Therefore, it is very important to understand the damaging effects of DBP on the soil ecosystem and soil health.

It is estimated that 1 g of black soil contains one billion microorganisms^11^. Soil microorganisms are considered as the key components for soil energy flow^7,12^ and are believed to be the major driving force of ecosystem functions^13,14^. Soil microorganisms are a significant part of the earth’s biodiversity and play key roles in carbon metabolism, nitrogen cycling and the overall functioning of an ecosystem^15,16^. Moreover, soil microorganisms play important roles in soil structure and development^17^. Changes in the microbial community structure not only alter the soil environment but also affect plant growth and soil fertility^18^. In the current studies, we applied metagenomics analysis and real-time fluorescent quantitative PCR (*q*PCR) to explore the impacts of DBP on the microbial ecology of black soils.

## Result

### DBP concentration in soils

**Figure 1:**
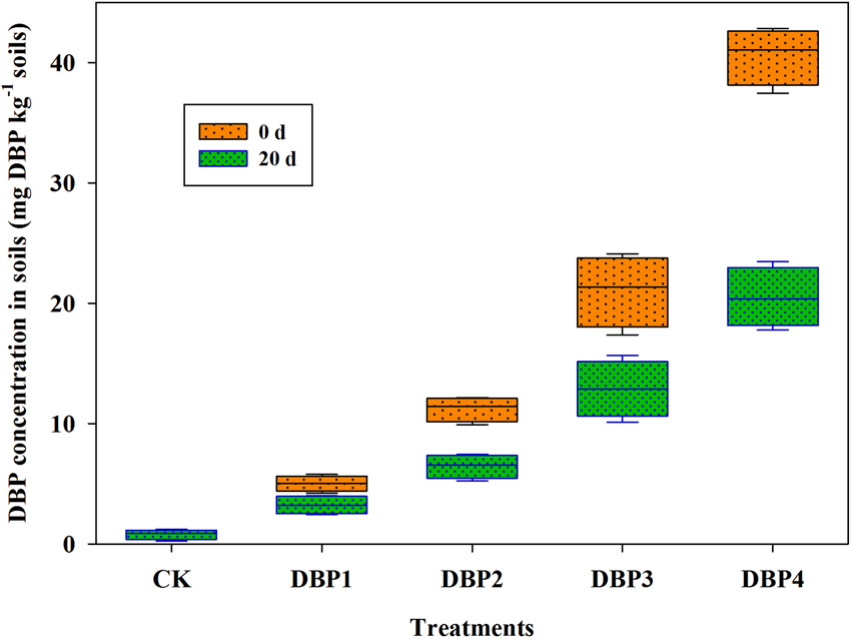
DBP concentration in soils from 0 to 40 mg kg^−1^ soil (CK, DBP1, DBP2, DBP3, and DBP4) in black soils incubated for 0 d and 20 days in the dark at 25 °C and 70% air humidity.

Figure 1 showed DBP concentration in black soils incubated for 0 d and 20 days. This result found that DBP concentration was consistent with the concentration of addition in experiment for 0 d (Fig. 1). Compare with 0 d, DBP concentration reduced in all samples of DBP treatment (DBP1, DBP2, DBP3 and DBP4) for 20 days. These results indicated that the residual rate of DBP was more than 50% at 20 days in black soils.

### Microbial community structure

**Figure 2:**
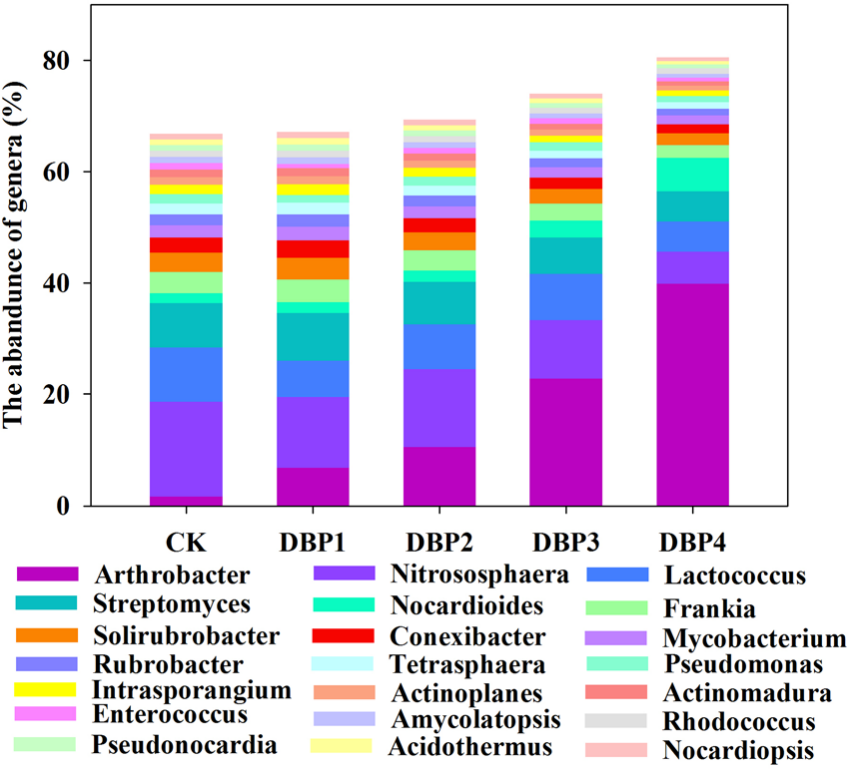
Comparisons among the community structures and the relative abundance of the 22 microbial genera revealed by metagenome analysis in black soils that were incubated in the dark at 25 °C and 70% air humidity for 20 days. The heatmap indicates the relative abundance of different genera in the community. The value (%) of relative abundance is represented by a color gradient.

The taxonomic profiles of the metagenome analysis, which contained domain, kingdom, phylum, class, order, family, genus and species, were constructed. With increasing DBP concentration, the percentages of Eukaryota were decreased from 2% to 0.9%, and the percentage of total bacteria was increased from 81% to 92%. We focused on the genus level to explain the changes in the microbial community. Based on the metagenome analysis, the results were shown in Fig. 2. The relative populations of the genera *Arthrobacter* and *Nocardioides* were increased along with the increase of DBP concentration. The relative abundances of *Arthrobacter* in CK, DBP1, DBP2, DBP3 and DBP4 were 1.67%, 6.82%, 10.56%, 22.82% and 39.86%, respectively. The relative abundance of *Nocardioides* in CK, DBP1, DBP2, DBP3 and DBP4 were 1.75%, 1.91%, 2.05%, 3.07% and 6.09%, respectively. On the other hand, the relative levels of some bacteria were decreased by DBP in all samples and showed a negative correlation with DBP concentration; these bacteria included *Nitrososphaera*, *Lactococcus*, *Streptomyces*, *Frankia*, *Solirubrobacter*, *Conexibacter*, *Mycobacterium*, *Rubrobacter*, *Tetrasphaera*, *Pseudomonas*, *Intrasporangium*, *Actinoplanes*, *Actinomadura*, *Enterococcus*, *Amycolatopsis*, *Rhodococcus*, *Pseudonocardia*, *Acidothermus*, *Nocardiopsis*, and others.

### Annotation of metabolic pathways by KEGG analysis

Metabolic pathways, including nitrogen cycling, carbon metabolism (glycolysis, TCA cycle and pentose phosphate), sulfur metabolism and signal regulatory pathways, were identified in DBP-contaminated black soils based on the KEGG database. The original data are shown in Supplementary Tables 1 and 2.

**Figure 3:**
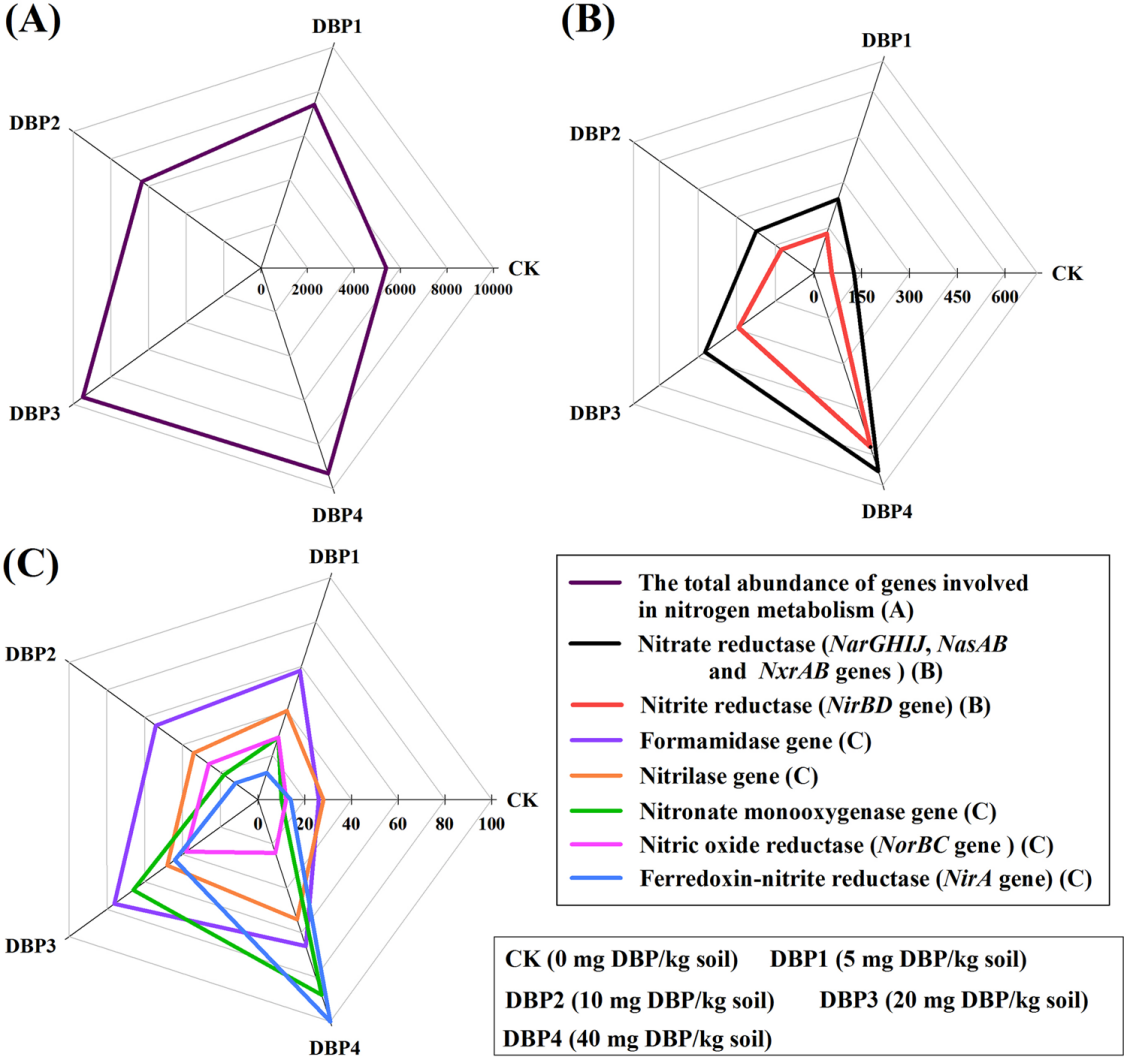
The abundances of nitrogen cycling genes under different DBP concentrations from 0 to 40 mg kg^−1^ soil (CK, DBP1, DBP2, DBP3, and DBP4) in black soils incubated for 20 days in the dark at 25 °C and 70% air humidity. (**A**) The total abundance of genes involved in nitrogen metabolism; (**B**) The abundances of nitrate reduction genes (*NarGHIJ*, *NasAB* and *NxrAB*) and the abundance of a nitrite reduction gene (*NirBD*); (**C**) The gene abundances of related enzymes involved in nitrogen cycling, such as formamidase, nitrilase, nitronate monooxygenase, ferredoxin-nitrite reductase and nitric oxide reductase.

As shown in Fig. 3, DBP contamination changed the flux of nitrogen cycling. The total abundance of nitrogen cycling genes rose significantly in response to the increase of DBP concentration (*F* = 25.71, *P* < 0.01) (Fig. 3A and Supplementary Table 7). Nitrite reductase (*NirBD*) (*F* = 234.76, *P* < 0.01), nitrate reductase (*NarGHIJ*, *NasAB* and *NxrAB*) (*F* = 136.06, *P* < 0.01), ferredoxin-nitrite reductase (*NirA*) (*F* = 188.95, *P* < 0.01) and nitric oxide reductase (*NorBC*) genes (*F* = 43.89, *P* < 0.01) are the key enzyme genes in nitrogen cycling, and their copy numbers were significantly increased after DBP contamination (Fig. 3B,C and Supplementary Table 7). The copy numbers of related enzyme genes in nitrogen cycling, such as the formamidase (*F* = 32.49, *P* < 0.01), nitrilase (*F* = 7.64, *P* < 0.05) and nitronate monooxygenase (*F* = 177.61, *P* < 0.01) genes, were increased significantly by DBP contamination (Fig. 3C and Supplementary Table 7).

**Figure 4:**
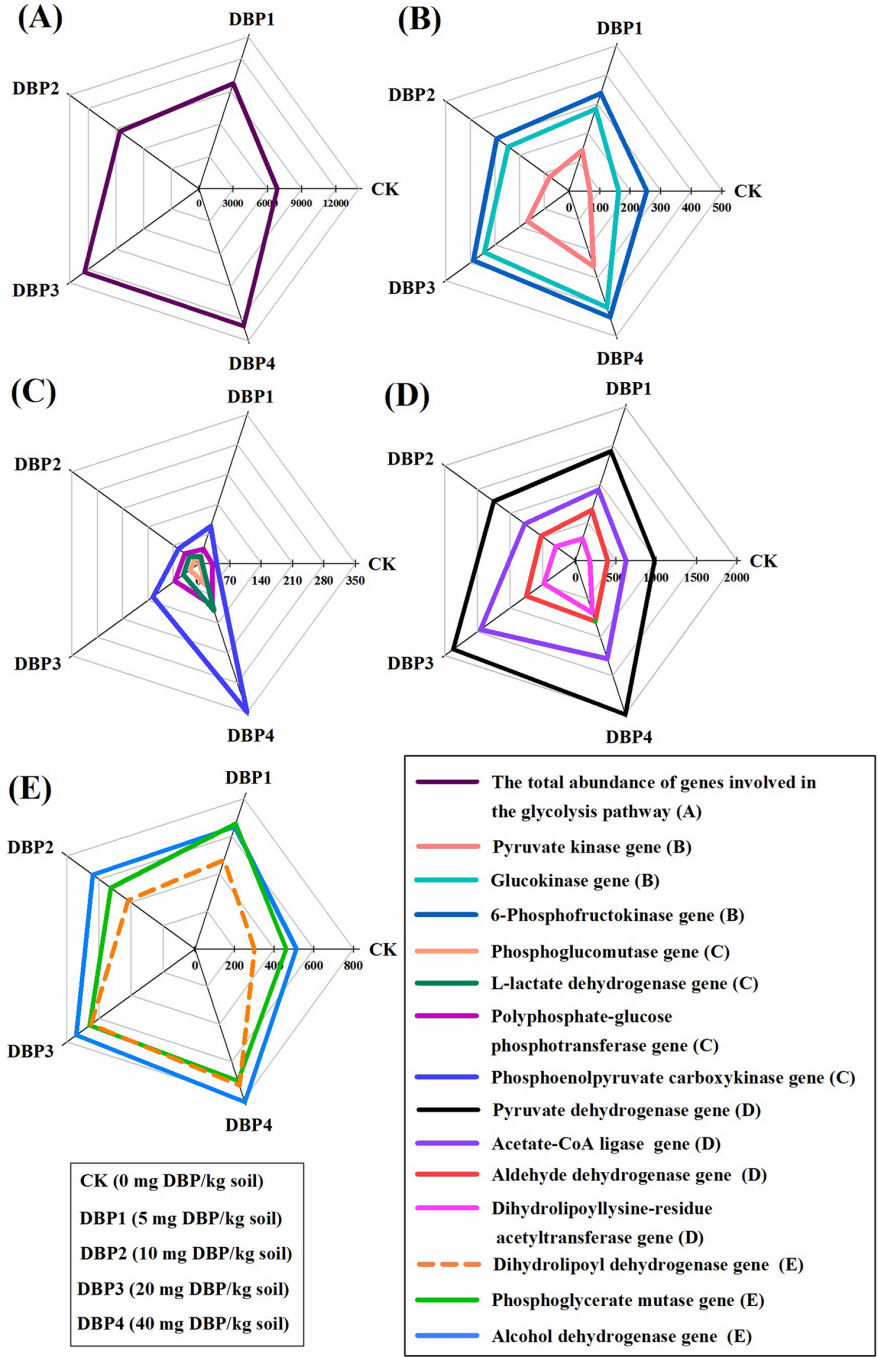
The abundances of glycolysis pathway genes under different DBP concentrations from 0 to 40 mg kg^−1^ soil (CK, DBP1, DBP2, DBP3, and DBP4) in black soils incubated for 20 days in the dark at 25 ^◦^C and 70% air humidity. (**A**) The total abundance of genes involved in the glycolysis pathway; (**B**) The gene abundances of the rate-limiting enzymes in the glycolysis; (**C**) and (**D**) The gene abundances of enzymes related to glycolysis.

**Figure 5:**
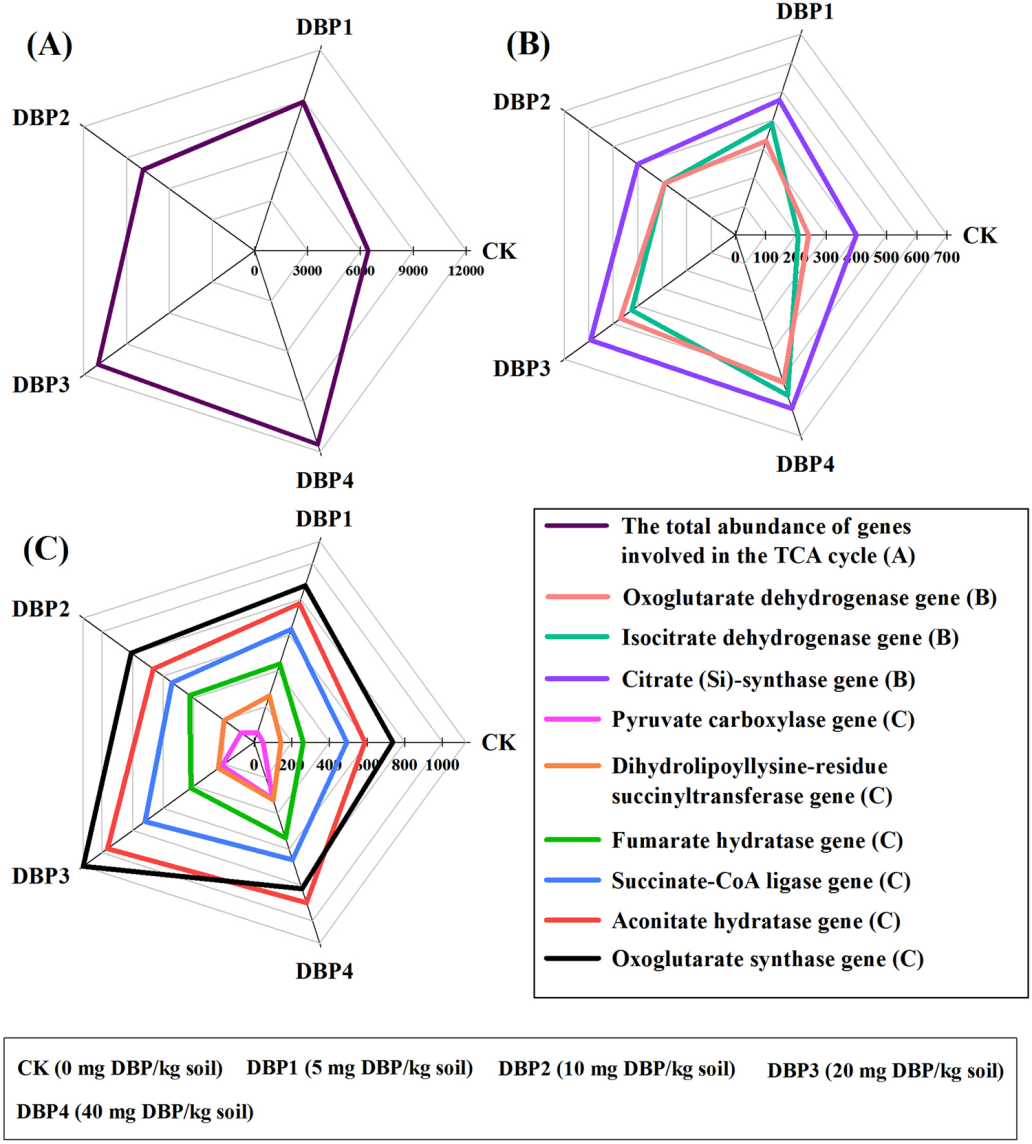
The abundances of TCA cycle genes under different DBP concentrations from 0 to 40 mg kg^−1^ soil (CK, DBP1, DBP2, DBP3, and DBP4) in black soils incubated for 20 days in the dark at 25 °C and 70% air humidity. (**A**) The total abundance of genes involved in the TCA cycle; (**B**) The gene abundances of the rate-limiting enzymes in the TCA cycle; (**C**) The gene abundances of enzymes related to the TCA cycle.

**Figure 6:**
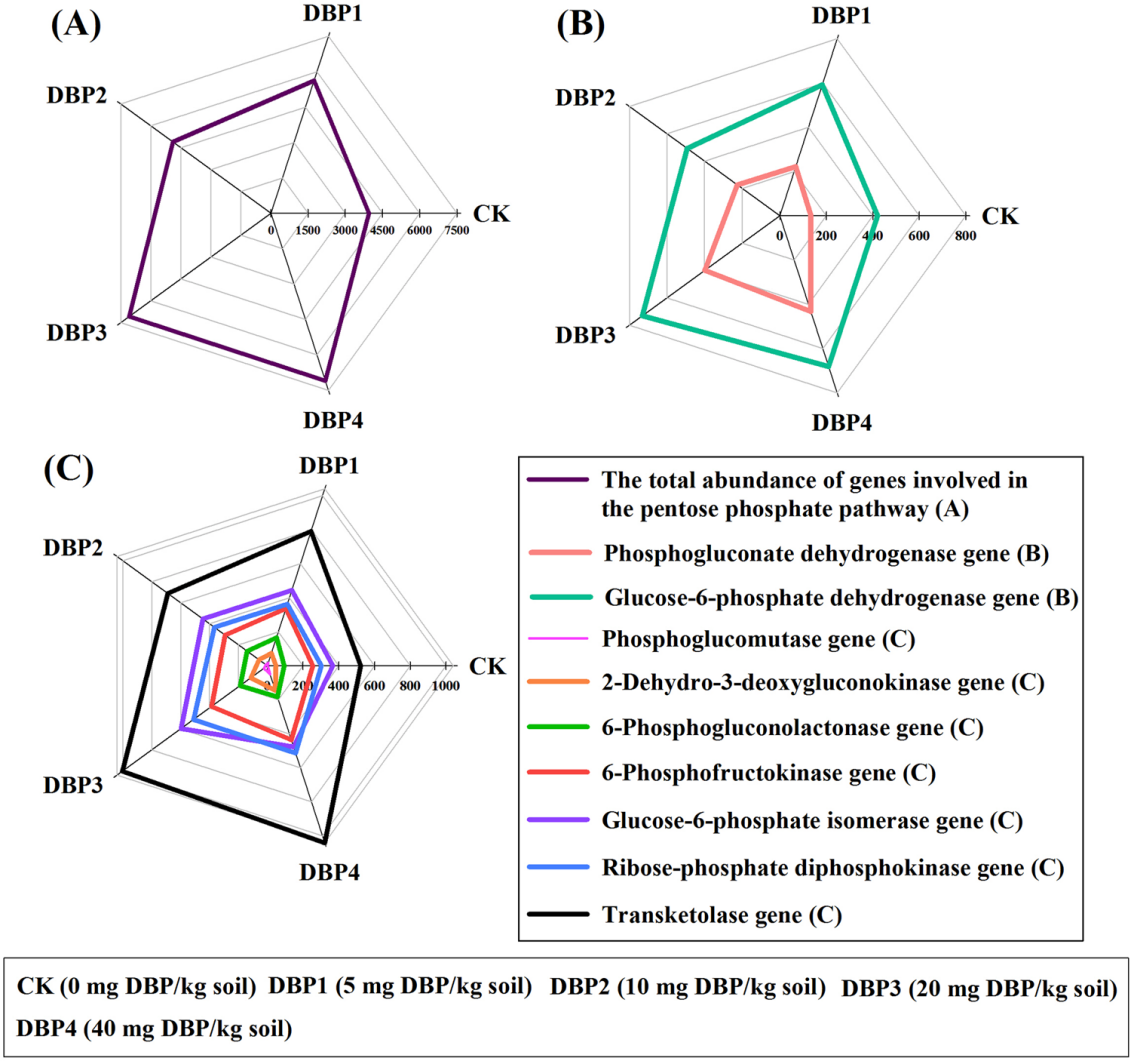
The abundances of pentose phosphate pathway genes under different DBP concentrations from 0 to 40 mg kg^−1^ soil (CK, DBP1, DBP2, DBP3, and DBP4) in black soils incubated for 20 days in the dark at 25 °C and 70% air humidity. (**A**) The total abundance of genes involved in the pentose phosphate pathway; (**B**) The gene abundances of the rate-limiting enzymes in the pentose phosphate pathway; (**C**) The gene abundances of enzymes related to the pentose phosphate pathway.

According to KEGG annotation and comparison of metagenome sequences, the flux of the carbon metabolic pathways, which included the glycolysis, TCA cycle and pentose phosphate pathways, were changed by DBP contamination (Fig. 4). As the DBP concentration increased, the total abundance of the glycolysis pathway genes was significantly enhanced in all samples of DBP treatments (*F* = 27.15, *P* < 0.01) (Fig. 4A and Supplementary Table 8). The gene abundances of the rate-limiting enzymes of glycolysis, which included pyruvate kinase (*F* = 109.34, *P* < 0.01), glucokinase (*F* = 11.83, *P* < 0.01) and phosphofructokinase (*F* = 17.16, *P* < 0.01), were also significantly promoted in sample of DBP treatments, including DBP1, DBP2, DBP3 and DBP4 (Fig. 4B and Supplementary Table 8). The gene abundances of enzymes related to glycolysis, which included phosphoglucomutase (*F* = 136.77, *P* < 0.01), L-lactate dehydrogenase (*F* = 71.74, *P* < 0.01), phosphoenolpyruvate carboxykinase (*F* = 242.13, *P* < 0.01), pyruvate dehydrogenase (*F* = 34.25, *P* < 0.01), acetate-CoA ligase (*F* = 49.49, *P* < 0.01), alcohol dehydrogenase (*F* = 13.41, *P* < 0.01), aldehyde dehydrogenase (*F* = 29.50, *P* < 0.01), dihydrolipoyl dehydrogenase (*F* = 46.40, *P* < 0.01), phosphoglycerate mutase (*F* = 12.50, *P* < 0.01) and dihydrolipoyllysine-residue acetyltransferase (*F* = 94.05, *P* < 0.01), were increased in all samples of DBP treatments (Fig. 4C–E and Supplementary Table 8). The gene abundance of polyphosphate-glucose phosphotransferase (*F* = 85.18, *P* < 0.01) was significantly increased in DBP of high concentration (DBP3 and DBP4) (Fig. 4C and Supplementary Table 8). In the TCA cycle, both the total gene abundance (*F* = 21.68, *P* < 0.01) and the gene abundance of the rate-limiting enzymes (including isocitrate dehydrogenase (*F* = 54.68, *P* < 0.01) and oxoglutarate dehydrogenase (*F* = 41.37, *P* < 0.01)) were raised in all samples of DBP treatments (Fig. 5A,B and Supplementary Table 9). The gene abundance of citrate synthase (*F* = 21.59, *P* < 0.01) had significance in in DBP of high concentration (DBP3 and DBP4) (Fig. 5B and Supplementary Table 9). The gene abundances of pyruvate carboxylase (*F* = 187.36, *P* < 0.01), dihydrolipoyllysine-residue succinyltransferase (*F* = 33.32, *P* < 0.01), fumarate hydratase (*F* = 8.34, *P* < 0.05), succinate-CoA ligase (*F* = 9.85, *P* < 0.01) and aconitate hydratase (*F* = 18.10, *P* < 0.01) were also increased (Fig. 5C and Supplementary Table 9). The gene abundance of oxoglutarate synthase (*F* = 11.89, *P* < 0.01) was significantly increased in DBP3 (Fig. 5C and Supplementary Table 9). The total gene abundance of the pentose phosphate pathway was positively correlated with DBP concentration (*F* = 25.65, *P* < 0.01) (Fig. 6A and Supplementary Table 10). The gene abundances of phosphogluconate dehydrogenase (*F* = 68.29, *P* < 0.01) and glucose-6-phosphate dehydrogenase (*F* = 21.90, *P* < 0.01), rate-limiting enzymes in the pentose phosphate pathway, were also augmented by DBP contamination in all samples of DBP treatments (Fig. 6B and Supplementary Table 10). The gene abundances of phosphoglucomutase (*F* = 190.05, *P* < 0.01), 2-dehydro-3-deoxygluconokinase (*F* = 97.44, *P* < 0.01), 6-phosphogluconolactonase (*F* = 25.85, *P* < 0.01), 6-phosphofructokinase (*F* = 19.09, *P* < 0.01), glucose-6-phosphate isomerase (*F* = 15.84, *P* < 0.01) and transketolase (*F* = 24.27, *P* < 0.01) were increased by DBP contamination in all samples of DBP treatments (Fig. 6C and Supplementary Table 10). The gene abundance of ribose-phosphate diphosphokinase was significantly enlarged in DBP of high concentration (DBP3 and DBP4) (*F* = 16.43, *P* < 0.01) (Fig. 6C and Supplementary Table 10).

**Figure 7:**
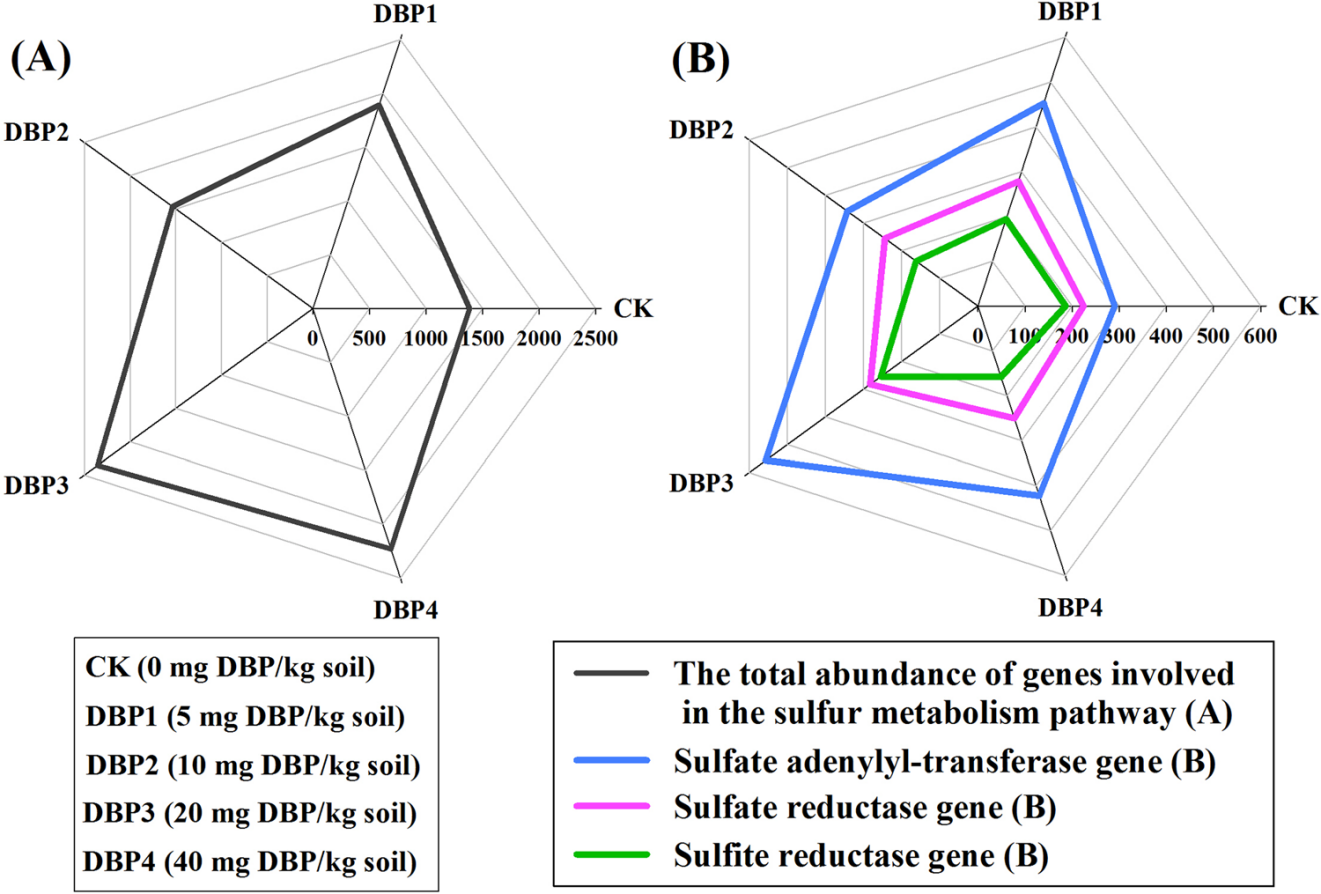
The abundances of genes in the sulfur metabolism pathway under different DBP concentrations from 0 to 40 mg kg^−1^ soil (CK, DBP1, DBP2, DBP3, and DBP4) in black soils incubated for 20 days in the dark at 25 °C and 70% air humidity. (**A**) The total abundance of genes involved in the sulfur metabolism pathway; (**B**) The gene abundances of the key enzymes in the sulfur metabolism pathway.

The total abundance of sulfur metabolism genes was higher in the contaminated samples than in CK (*F* = 21.69, *P* < 0.01) (Fig. 7A and Supplementary Table 11). The gene abundance of sulfate adenylyl-transferase (*F* = 22.91, *P* < 0.01), which included *Sat*, *CysND* and *CysC*, was promoted to various extents with increasing DBP concentration (Fig. 7B). The gene abundances of sulfite reductase (*F* = 18.78, *P* < 0.01) was significantly enlarged in DBP1 and DBP3, but it was decreased in DBP2 and DBP4 (Fig. 7B and Supplementary Table 11). The gene abundance of sulfate reductase (*F* = 3.83, *P* < 0.05) was enhanced in all samples of DBP treatments (Fig. 7B and Supplementary Table 11).

### Signal regulatory pathways

**Figure 8:**
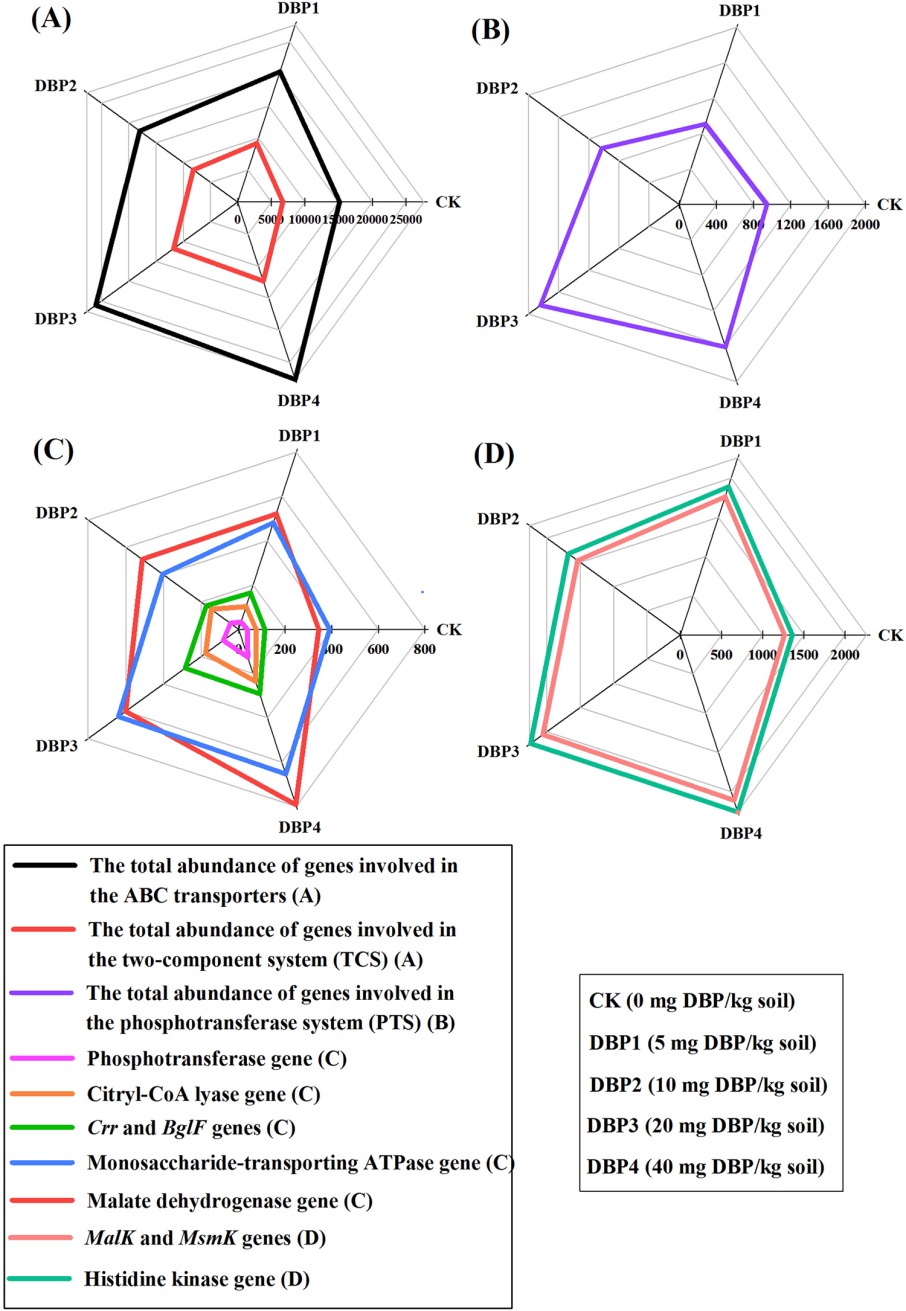
The gene abundances of the signal regulatory pathways under different DBP concentrations from 0 to 40 mg kg^−1^ soil (CK, DBP1, DBP2, DBP3, and DBP4) in black soils incubated for 20 days in the dark at 25 °C and 70% air humidity. (**A**) The total abundance of genes involved in the ABC transporters and two-component system (TCS); (**B**) The total abundance of genes involved in the phosphotransferase system (PTS); (**C**) and (**D**) The gene abundances of enzymes related to the ABC transporters, TCS and PTS.

The total gene abundances of the ABC transporters (*F* = 27.45, *P* < 0.01) and two-component system (TCS) (*F* = 25.81, *P* < 0.01) were promoted in all samples of DBP treatments (Fig. 8A and Supplementary Table 12). The total gene abundances of the phosphotransferase system (PTS) were increased in DBP3 and DBP4 (*F* = 44.73, *P* < 0.01) (Fig. 8B and Supplementary Table 12). The gene abundances of the monosaccharide-transporting ATPases (*F* = 31.22, *P* < 0.01), *MalK* and *MsmK* (*F* = 17.95, *P* < 0.01) of the ABC transporters were increased under DBP contamination (Fig. 8C and D and Supplementary Table 12). In the TCS, DBP contamination brought about an increase in the gene abundances of malate dehydrogenase (*F* = 23.12, *P* < 0.01), histidine kinase (*F* = 18.06, *P* < 0.01) and citryl-CoA lyase (*F* = 75.29, *P* < 0.01) (Fig. 8C,D and Supplementary Table 12). The gene abundances of the phosphotransferases (*F* = 116.26, *P* < 0.01), *Crr* and *BglF* (*F* = 67.57, *P* < 0.01) in the PTS were significantly increased by DBP and were positively correlated with DBP concentration (Fig. 8C and Supplementary Table 12).

### Nonmetric multidimensional scaling (NMDS) analysis

**Figure 9:**
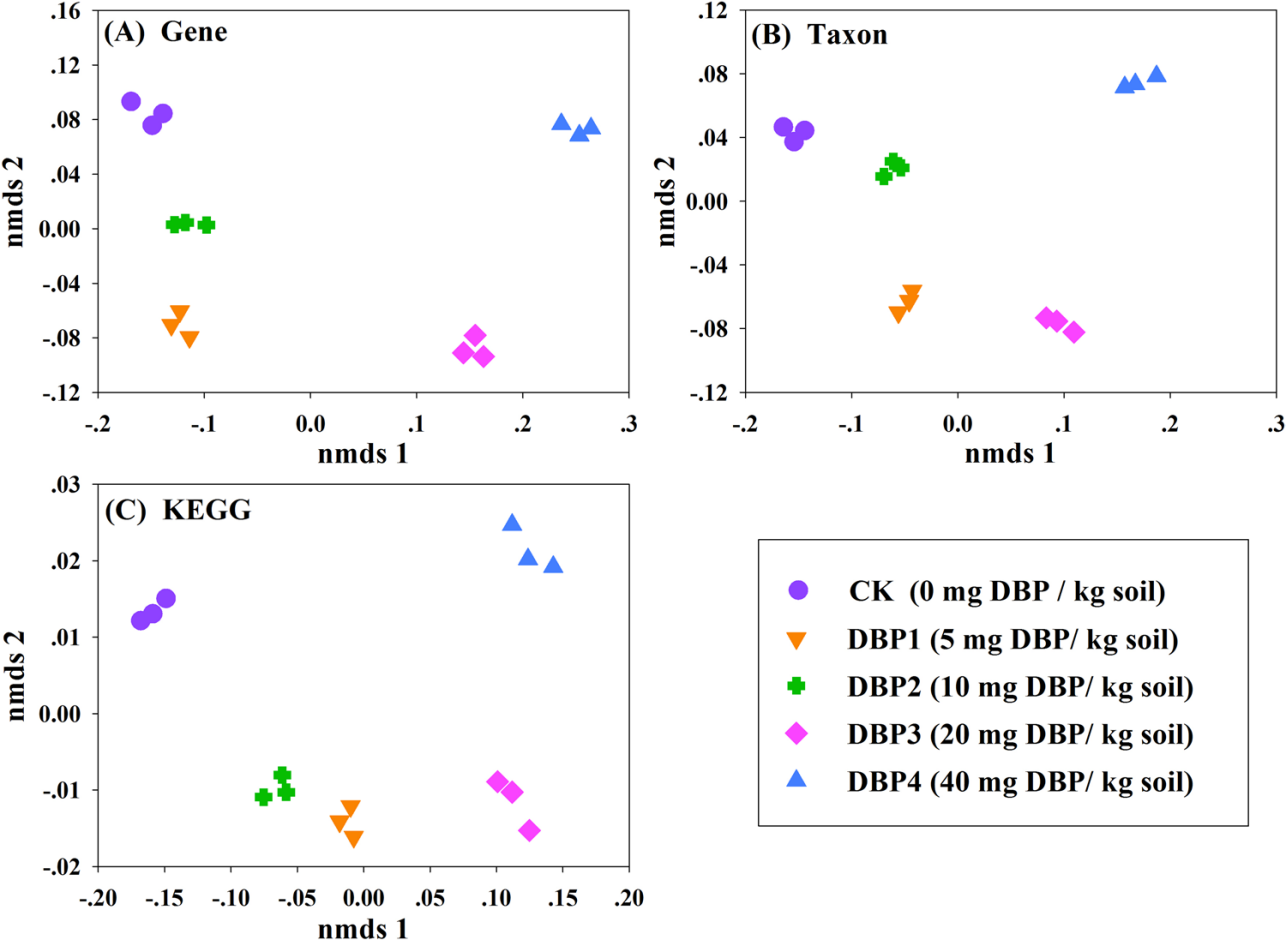
NMDS analysis on various levels among control and DBP-treated samples (CK, DBP1, DBP2, DBP3, and DBP4) in black soils incubated for 20 days in the dark at 25 °C and 70% air humidity. (**A**) NMDS analysis on the gene level; (**B**) NMDS analysis on the taxon level; (**C**) NMDS analysis on the KEGG pathway level.

To explain the overall differences among the CK and DBP contamination samples, NMDS analysis was performed at the levels of genes, taxonomy and metabolic pathways. The results indicated that DBP contamination altered the distribution of genes, taxons and metabolic genes (Fig. 9). Furthermore, the difference became larger with increasing DBP concentrations.

### DBP degradation genes

**Figure 10:**
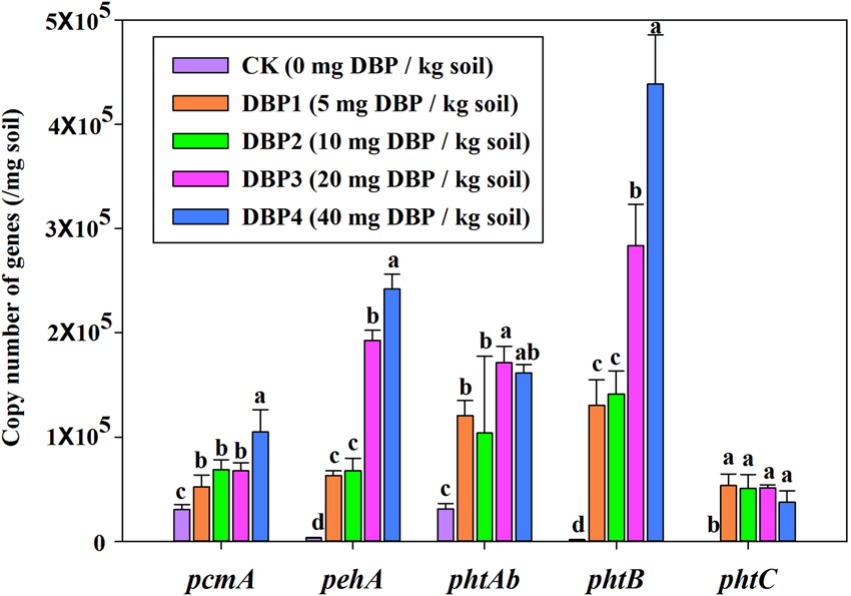
Gene abundances of *pehA*, *phtB*, *phtAb*, *pcmA* and *phtC* based on qPCR analysis under various DBP concentrations varied from 0 to 40 mg kg^−1^ soil (CK, DBP1, DBP2, DBP3, and DBP4) in black soils incubated in the dark at 25 °C and 70% air humidity for 20 days.

The main DBP degradation genes are *pcmA*, *pehA*, *phtAb*, *phtB* and *phtC*. The copy numbers of *pcmA*, *pehA*, *phtAb*, *phtB* and *phtC* were significantly increased under DBP contamination (Fig. 10). When the concentration of DBP increased, the copy numbers increased as well, and the range of increase varied. The lowest degree of change was that of *pcmA*, which increased from 3.06 × 10^4^ to 1.05 × 10^5^. The greatest change was that of *phtB*, which increased from 1.55 × 10^3^ to 4.39 × 10^5^. Additionally, the copy numbers of *pehA* and *phtAb* genes were elevated along with increasing DBP concentration, increasing from 3.29 × 10^3^ to 2.42 × 10^5^ and from 3.13 × 10^4^ to 1.62 × 10^5^, respectively. After DBP contamination, the copy number of *phtC* was higher compared with CK, increasing from 94.49 to 5.35 × 10^4^. The results were consistent with the change of DBP concentration in black soil (Fig. 1).

## Discussion

Using metagenomic analysis, this study found that the microbial community structure was changed in black soil under DBP contamination in the short term. The abundances of some genera, such as *Arthrobacter* and *Nocardioides*, were significantly enriched in response to DBP treatment. Previous studies showed that *Arthrobacter* and *Nocardioides* could degrade organic contamination^19,20^. This phenomenon could be further demonstrated by comparing the copy numbers of DBP degradation genes (Fig. 10). However, the growth for some genera that are indispensable for nutrient cycling^21–23^ were inhibited by DBP, including *Nitrososphaera*, *Streptomyces*, *Frankia*, *Conexibacter*, *Pseudonocardia*, *Acidothermus*, *Nocardiopsis*, *Lactococcus*, *Mycobacterium*, *Pseudomonas* and *Tetrasphaera*. *Nitrososphaera*, *Frankia* and *Conexibacter*. *Streptomyces*^24,25^, *Conexibacter*^26^, *Pseudonocardia*^27^, *Acidothermus*^28^, *Nocardiopsis*^29^, *Lactococcus*^30^, *Mycobacterium*^31,32^, *Pseudomonas*^33^ and *Tetrasphaera*^34^ are beneficial to soil health and plant growth. *Amycolatopsis* and *Intrasporangium* were also decreased even though they could remediate heavy metal pollution^35,36^. The changes in the microbial community indicated that DBP contamination disturbed the soil ecosystem in the short term.

Nitrogen cycling, carbon metabolism and sulfur metabolism are the basis of nutrient cycling in soil ecosystems^37–39^. The turnover of the nitrogen cycle, the glycolysis pathway, the TCA cycle, the pentose phosphate pathway and sulfur metabolism were accelerated significantly when soil microorganisms were exposed to DBP contamination. In nitrogen cycling, the genes *NarGHIJ*, *NasAB*, *NxrAB*, *NirBD*, and *NorBC* are the key genes to the dissimilatory nitrate reduction, assimilatory nitrate reduction, denitrification and nitrification, and their abundances were elevated due to DBP contamination. Meanwhile, the gene abundances of *NorBC* and *NarGHIJ* were increased. Because *NorBC* and *NarGHIJ* catalyze the generation of nitrous oxide (N_2_O) and nitrite^40^, nitrite accumulation and N_2_O emission could be increased. In this study, it was found that the turnover of the carbon metabolic pathways was accelerated after DBP treatment such that more carbons in the substrate were converted to CO_2_, which meant that more carbon could be consumed in the soils. It was also found that the gene abundance of sulfate adenylyltransferase was increased in the soil microorganisms. The phenomenon implied that DBP contamination facilitated 3′-phosphoadenosine-5′- phosphosulfate (PAPS) formation. This result could indicate that the flux of sulfate metabolism was increased and that more sulfate turned toward the direction of oxidative metabolism after DBP contamination^39,41,42^. For all of these results, the metabolic processes of soil microorganisms were accelerated including nitrogen cycling, carbon metabolism and sulfur metabolism, which could affect the nutrient transformation and the quality of black soils in the short term.

ABC transporters are found in all domains of organisms and transfer a remarkably wide range of substrates into and out of living cells^43,44^. ABC transporters are useful for transporting substrates including sugars, amino acids, peptides, ions and other molecules as well as for absorbing nutrients into cells^45,46^. Increases in the total abundance of ABC transporters and the relative gene abundances of the monosaccharide-transporting ATPases *MalK* and *MsmK* could be the reason for the accelerated metabolism of nitrogen, carbon and sulfate, which may accelerate the depletion of nutrients in black soils. In prokaryotes, TCS is a sophisticated signal transduction strategy and senses any alteration in the environment^47^. It regulates downstream gene expression, thus orchestrating several cellular responses, and participates in biochemical processes, acting as an energy carrier^48^. The total abundance of TCS genes and the abundances of the malate dehydrogenase, histidine kinase and citryl-CoA lyase genes were increased after DBP contamination, suggesting that it could lead to accelerated metabolism in black soils, including nitrogen, carbon and sulfate metabolism. PTS is the key signal transduction pathway involved in the regulation of central carbon and nitrogen metabolism in bacteria^49,50^. The total abundance of PTS genes and the gene abundances of phosphotransferase, *Crr* and *BglF* were raised by DBP. These manifestations indicated that the increase of PTS was one of the causes of the acceleration of nitrogen and carbon metabolism. Hence, these changes of ABC transporters, TCS and PTS brought about metabolic changes in black soils in the short term.

## Conclusion

Based on the results of metagenome analysis and qPCR analysis in the short term (20 days), we propose that DBP contamination altered the structure of the microbial community; improved the gene abundances of nitrogen cycling, glycolysis, the TCA cycle, the pentose phosphate pathway and sulfate metabolism; and increased the abundance of DBP degradation genes. The increased abundances of signal regulatory pathways for ABC transporters, TCS and PTS could be the reason for the changes of gene abundances in carbon, nitrogen and sulfur metabolism. However, it remains to be seen whether structure and function are impacted over the long-term.

## Materials and Methods

### Sample collection and treatment

DBP (chromatographically pure grade) was purchased from the Information Center for Standard Samples (Beijing, China). Acetone (chromatographically pure grade) was acquired from Traditional Chinese Medicine (Beijing, China). DBP was dissolved in acetone at a ratio of 1 to 9 (m/m) and then stored in the dark at 4 °C.

The black soil samples were collected randomly from various areas of Keshan County of Heilongjiang Province in China, which had no history of DBP contamination as of August 5, 2015. All soils were under the same crop, and then excavating soils 200 kg within 20 cm of the black soil surface after removing the top surface layer in different locations. The physicochemical properties of the black soils are shown in Table 1. Soils were sieved by 0.3 mm mesh. After blending and sieving, the soils were divided into 15 samples of 1000 g each. Afterwards, the soil was moistened up to 30% and preincubated at 25 °C for 7 days. Varied amounts of DBP were added to the soil samples and mixed well, as follows: Control (CK), 0 mg DBP kg^−1^ soil; DBP sample 1 (DBP1), 5 mg DBP kg^−1^ soil; DBP sample 2 (DBP2), 10 mg DBP kg^−1^ soil; DBP sample 3 (DBP3), 20 mg DBP kg^−1^ soil; and DBP sample 4 (DBP4), 40 mg DBP kg^−1^ soil. The measured concentrations of DBP from the different treatments are shown in the Supplementary Figure 1. Each concentration stress was repeated 3 times. All the samples were exposed to the air for 3 h until the acetone was evaporated completely. The soil samples were placed in porcelain pots and incubated in the dark at 25 °C for 20 days. During the period of experiment, all samples were maintained at 30% moisture. DBP concentrations were measured in black soils incubated for 0 d and 20 days in the dark at 25 °C and 70% air humidity in constant temperature and humidity incubator.

**Table 1:** Physicochemical properties of black soils obtained from northeast China.

Soil type	Textural class (USDA)	pH	Organic matter (OM) g kg^−1^	TN g kg^−1^	TP g kg^−1^	Available phosphorus mg kg^−1^	Available potassium mg kg^−1^
Mollisols	Silty clay loam	6.04 ± 0.11	42.14 ± 8.54	2.56 ± 0.62	1.54 ± 0.38	24.21 ± 3.24	208.64 ± 29.54

### DMP concentration in soil

First, DBP of different samples were extracted using rotary evaporators. Then, DBP concentrations were measured by liquid chromatography for 0 d and 20 days. Parameters of liquid chromatography: sample size = 1 μl; Chromatographic column was 5 μl Eclipse XDB-C18; The chromatographic conditions: the temperature of the column = 25 °C, flow phase: methanol and water, volume ratio = 90:10; Detection wavelength = 228 nm.

### DNA extraction, metagenomic sequencing and paired-end (PE) library construction

Ten grams of soil were taken out from each pot. Then, the total DNA was extracted from the samples and used for the metagenome sequencing and further analyses^51^. E.Z.N.A.® Soil DNA Kit was used to extract the soil DNA (Supplementary Table 3). DNA concentration, purity, and integrity were detected by NanoDrop2000, TBS-380 and agarose gel electrophoresis, respectively. 1 μg DNA was used to build library. Briefly, the DNAs were sheared using an M220 Focused-ultrasonicator™ (Covaris Inc., Woburn, MA, USA) and the parameters of instrument were set according to Supplementary Table 4. The PE library was composed of ~300 bp DNA fragments. The TruSeq® DNA Sample Prep Kit was used to prepare DNA library based on the manufacturer’s protocol (www.illumina.com). Primer hybridization sites were included in dual-index adapters and ligated to the blunt-end fragments. To improve quality of the template DNA, paired-end sequencing (2 × 151 bp) was conducted on an Illumina Genome Analyzer (Illumina Inc., USA) by Majorbio Bio-Pharm Technology Co., Ltd. (Shanghai, China). All the raw metagenomic datasets have been deposited into the NCBI Sequence Read Achieve (accession numbers: SRP094732, SRP096214).

### Sequence quality control, assembly and gene prediction

To improve the quality and reliability of subsequent analysis, the raw data were processed using the following steps:

1. SeqPrep (https://github.com/jstjohn/SeqPrep) was used to remove the reads that contained N bases in the sequence and strip the 3′-adaptors and 5′-adaptors.
2. To retain high-quality pair-end reads and single-end reads, the reads of sequence length <50 bp and quality score <20 were excised with Sickle (https://github.com/najoshi/sickle). Clean reads were assembled with SOAPdenovo (http://soap.genomics.org.cn,Version 1.06) with a default k-mer length (39–47), and the de Bruijn graphs were used to build contigs. For the optimal assembly, scaffolds over 500 bp were divided into new contigs without gaps. The open reading frames (ORFs) of the contigs from the assembled results were predicted using MetaGene Annotator (http://metagene.cb.k.u-tokyo.ac.jp/).

### Taxonomy and functional annotation

The predicted ORFs of ≥100 bp were selected and translated to amino acid sequences by the NCBI translation table (http://www.ncbi.nlm.nih.gov/Taxonomy/taxonomyhome.html/index.cgi?chapter = tgencodes#SG1), and the sequences were subsequently annotated by searching BLASTP (Version 2.2.28+, http://blast.ncbi.nlm.nih.gov/Blast.cgi) against the NCBI NR database containing SwissProt, PRF (Protein Research Foundation), PIR (Protein Information Resource), PDB (Protein Data Bank), and protein information resources from corresponding coding sequences (CDS) on RefSeqG and GenBank. The BLAST parameters were set as follows: E-value cutoff = 1*10^−5^, num_alignments = 250, and num_descriptions = 500.

The taxonomy annotation was obtained based on the taxonomic information database corresponding to the NCBI NR library. The species abundance was calculated using the total gene abundance across all species, and the abundance profile was built based on taxonomic levels, which were the domain, kingdom, phylum, class, order, family, genus and species.

The KEGG database (Kyoto Encyclopedia of Genes and Genomes, http://www.genome.jp/kegg/) is a huge library for systematic analysis of gene function, combining genome and function information. It provides sequence information on the genes and proteins identified in genome projects. Further, the database includes various pathways, such as metabolic, biosynthetic, membrane transport, signaling, cell cycle and disease pathways. In addition, it collects records of all kinds of enzymes and other molecules as well as enzymatic reactions and related information. The KEGG pathway annotation was done using KOBAS 2.0 (KEGG Orthology Based Annotation System, http://kobas.cbi.pku.edu.cn/home.do) according to the alignment results between the gene set sequence and the KEGG database using BLAST (BLAST Version 2.2.28+, http://blast.ncbi.nlm.nih.gov/Blast.cgi). The amassed read data for each sample are presented in Supplementary Table 5.

### Nonmetric multidimensional scaling (NMDS)

Nonmetric multidimensional scaling (NMDS) is a monotonic function that evaluates the similarities or differences of different samples based on distance. In this study, NMDS represented the overall difference between control and DBP (varied concentration) contamination samples in taxonomy, genes and KEGG pathways. NMDS was generated by R language release package (MASS package).

### Real-time fluorescent quantitative PCR (*q*PCR)

*q*PCR was performed to detect the genes associated with the degradation of DBP, including phthalate ester hydrolase (*pehA*), 3,4-dihydrophthalate dehydrogenase (*phtB*), phthalate dioxygenase small subunit (*phtAb*), protocatechuate 4,5-dioxygenase (*pcmA*) and 3,4-dihydrophthalate decarboxylase (*phtC*)^52^. The primer information for these genes is shown in Table 2. PCR was conducted in 20-μL reactions containing 10 μL of 2 × SYBR green qPCR master mix (premix of dNTPs, Taq DNA polymerase, PCR buffers and SYBR green), 0.4 μL of primer F and 0.4 μL of primer R of each gene-specific primer pair, 7.2 μL of ddH_2_O and 2 μL of template (DNA) based on the manufacturer’s instructions (LightCycler480, Roche). DNA concentration was shown in Supplementary Table 6. The *q*PCR thermal cycling steps are shown in Table 3. The melting curves for the genes were obtained during the detection, representing the specificity of the amplification. A linear relationship was shown based on standard curves ranging from 10^3^ to 10^10^ copies. The amplification efficiencies ranged from 71.2% to 107.8%, and R^2^ ranged from 0.9901 to 0.9984 (Supplementary Figure 2). Analyses of variance (ANOVA) and the least significant ranges test (Duncan’s method) were performed with SPSS 22.0 to test the significance (*p* < 0.05) of the differences among the treatments.

**Table 2:** DBP degradation genes and relevant primers.

	*pehA*	*phtAb*	*phtB*	*phtC*	*pcmA*
Gene product	Putative phthalate ester hydrolase	Phthalate dioxygenase, small subunit	3,4-Dihydroxy-3,4-dihydrophthalate dehydrogenase	3,4-Dihydroxyphthalate decarboxylase	Protocatechuate 4,5- dioxygenase
Primer-F	5′-TGAGATGCTGCTCGTGAAGAA-3′	5′-AGACAACCCGAACGAAATCA-3′	5′-AACAAGGAGTCCCACAGCATT-3′	5′-GATACTCCGCCCCTAACGAA-3′	5′-TCCATGTGCTGCCATTCTGT-3′
Primer-R	5′-AGATAGGTGTTTGCCTCGTCC-3′	5′-ATAACCGACTCATCCACCATG-3′	5′-TCCAATAACTTCACGAGTCCCT-3′	5′-ACGACAGGACGATCACCCAT-3′	5′-CGAGCCGCTCAAAGTTCAA-3′
Product Length	279 bp	168 bp	158 bp	265 bp	153 bp

**Table 3:** qPCR thermal cycling steps.

Thermal cycler	Times and temperatures			
	Initial Steps	Each of 40 cycles		
		Melt	Anneal	Extend
Light Cycler 480 Software Setup(Roche)	hold	cycle		
	3 min 95 °C	15 s 95 °C	20 s 57 °C	30 s 72 °C

## Electronic supplementary material

Supplementary Information
