# Supplementary material for: Dibutyl phthalate alters the metabolic pathways of microbes in black soils
Source: Sci Rep. 2018 Feb 8;8:2605. doi: 10.1038/s41598-018-21030-8 (PMC5805725; doi:10.1038/s41598-018-21030-8)
Supplement: Supplementary file 1 — Supplementary Information [file 41598_2018_21030_MOESM1_ESM.pdf]

Dibutyl phthalate alters the metabolic pathways of microbes in black soils

Weihui Xu[1], Yimin You[1], Zhigang Wang[1,2*], Wenjing Chen[1], Jin Zeng[3], Xiaosong Zhao[1], Yunpeng Su[1]

[1]School of Life Science and Agriculture and Forestry, Qiqihar University, Qiqihar, Heilongjiang, 161006, China. [2]Institute for Environmental Genomics, Department of Microbiology and Plant Biology, University of Oklahoma, Norman, OK, 73072, USA. [3]State Key Laboratory of Lake Science and Environment, Nanjing Institute of Geography and Limnology, Chinese Academy of Sciences, Nanjing, 210000, China.

**Correspondence and requests for materials should be addressed to* Z.G.W. (e*mail: wzg1980830@sina.com*)

W.H.X. (e*mail: xwh800206@163.com*), Y.M.Y. (e*mail: strivingmin@163.com*), W.J.C. (e*mail: 1528799025@qq.com*), J.Z. (e*mail:* 147679352@qq.com), X.S.Z. (e*mail: zxiaosong1993@163.com*), Y.P.S. (e*mail: 313189237@qq.com*)

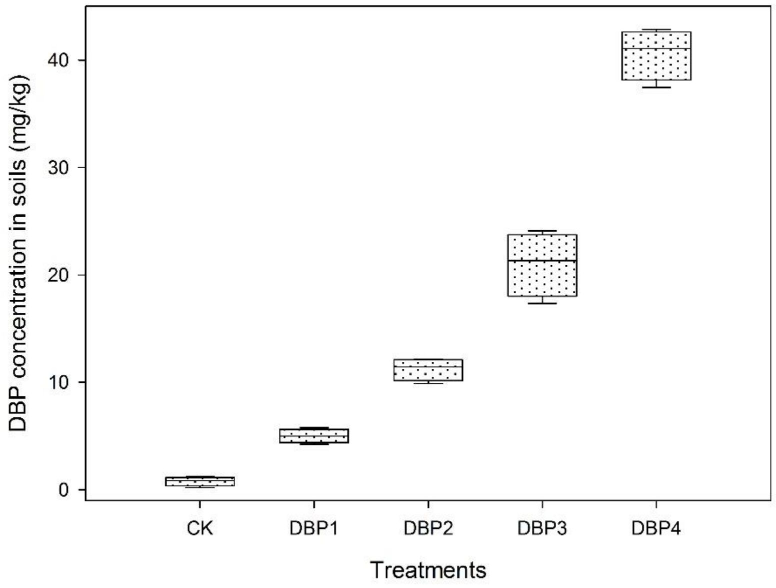

Supplementary Figure 1. The measured initial concentration of DBP in soils

Supplementary Figure 2. The amplification curves, melting peaks and standard curves of the degradation genes

pcmA:

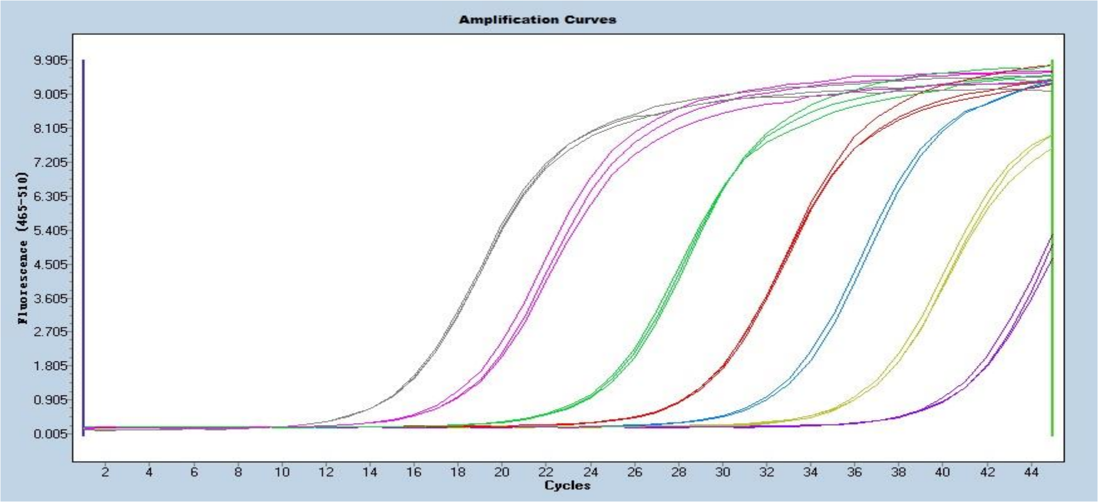

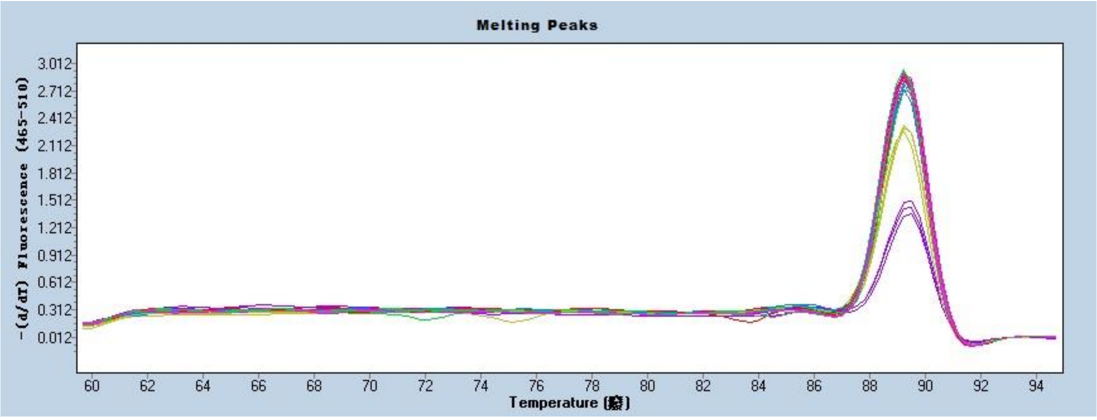

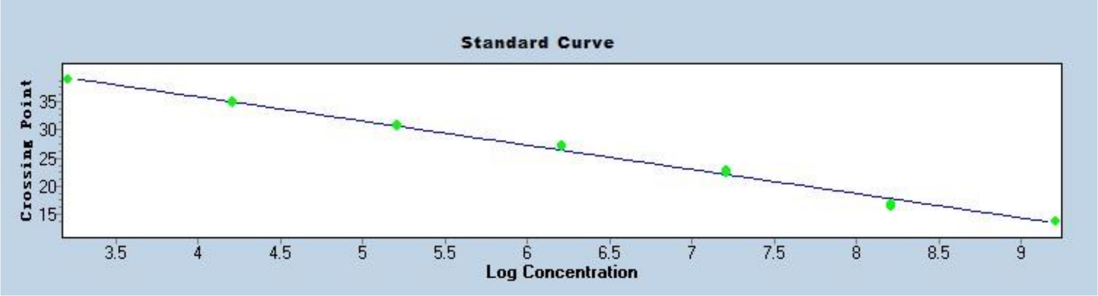

Note: Ordinate is CT；abscissa LogCO，LogCO is Log concentration (sample concentration of logarithm)

Slope=-4.281

Amplification efficiency: $E=10^{-1/slope}-1=10^{-1/-4.281}-1=71.2\%$

Correlation coefficient: $R^2=0.9954$

Y Intercept: 52.96

Error: 0.203

pehA:

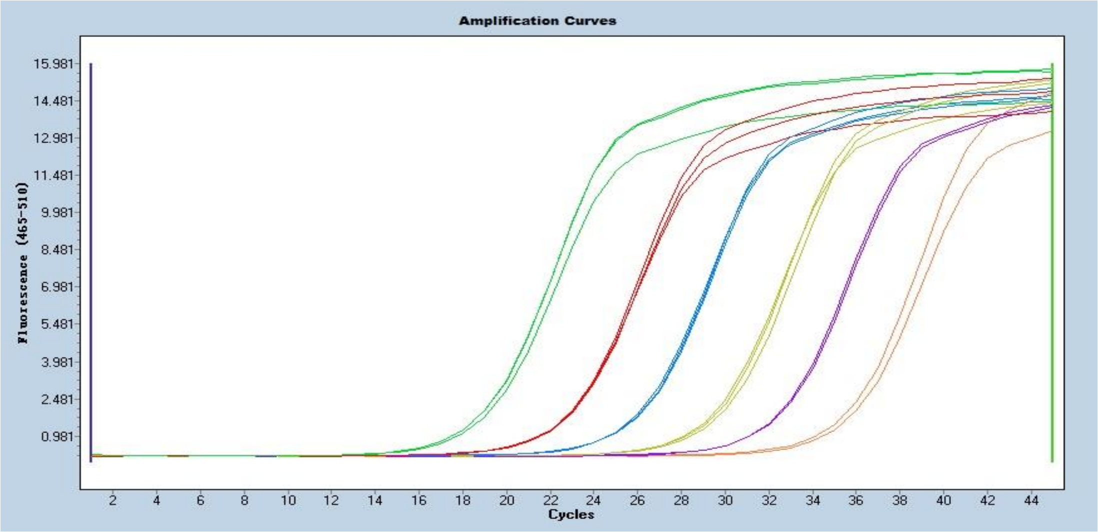

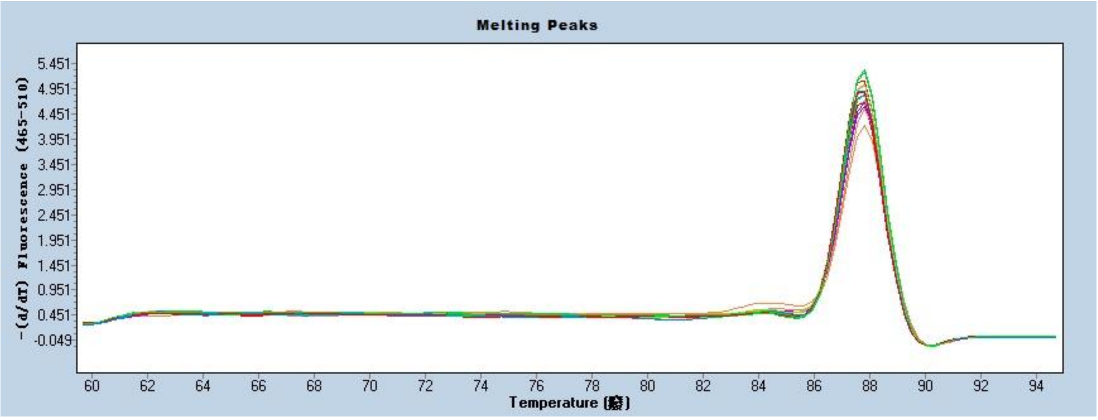

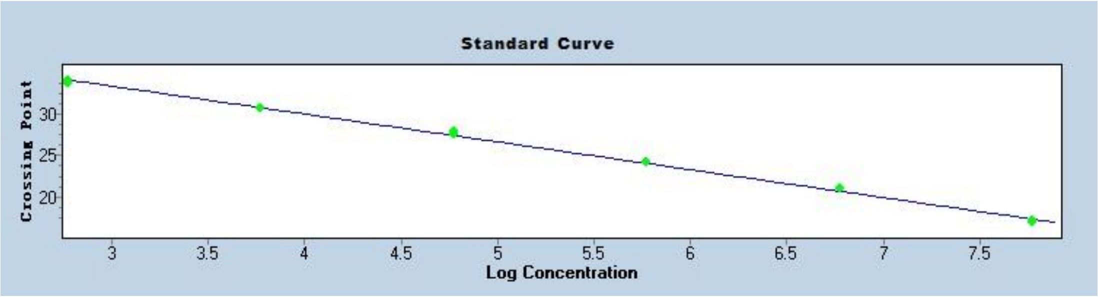

Note: Ordinate is CT；abscissa LogCO，LogCO is Log concentration (sample concentration of logarithm)

Slope=-3.307

Amplification efficiency: $E=10^{-1/slope}-1=10^{-1/-3.307}-1=100.6\%$

Correlation coefficient: $R^2=0.9976$

Y Intercept: 43.15

Error: 0.101

PhtAb:

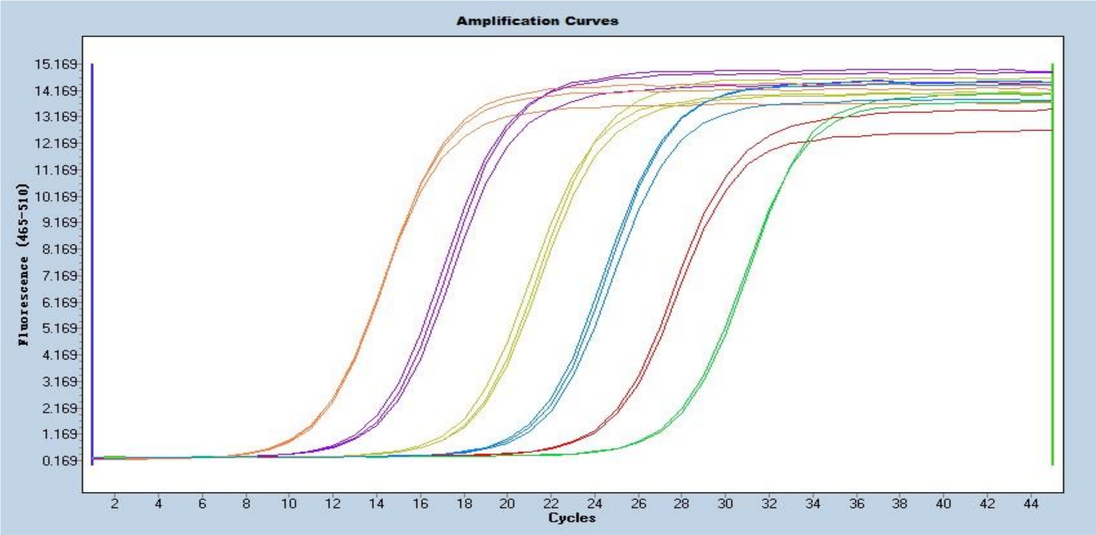

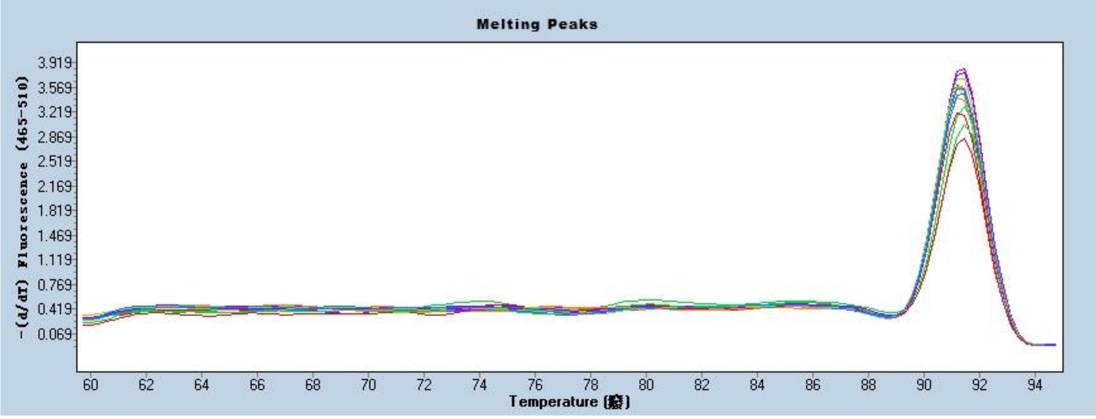

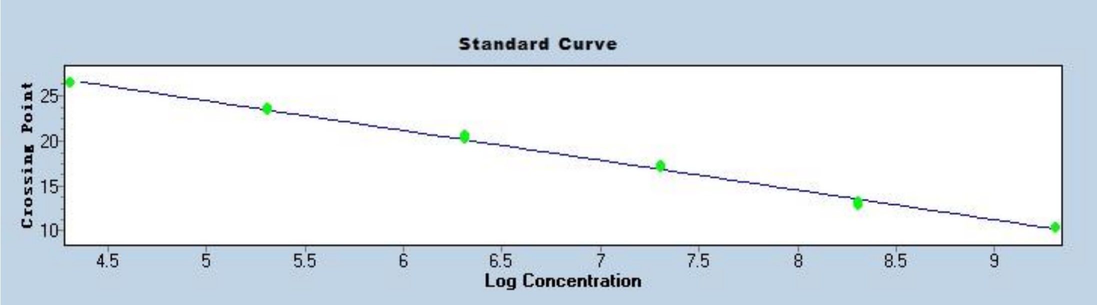

Note: Ordinate is CT；abscissa LogCO，LogCO is Log concentration (sample concentration of logarithm)

Slope=-3.301

Amplification efficiency: $E=10^{-1/slope}-1=10^{-1/-3.301}-1=100.9\%$

Correlation coefficient: $R^2=0.9965$

Y Intercept: 40.94

Error: 0.118

phtB:

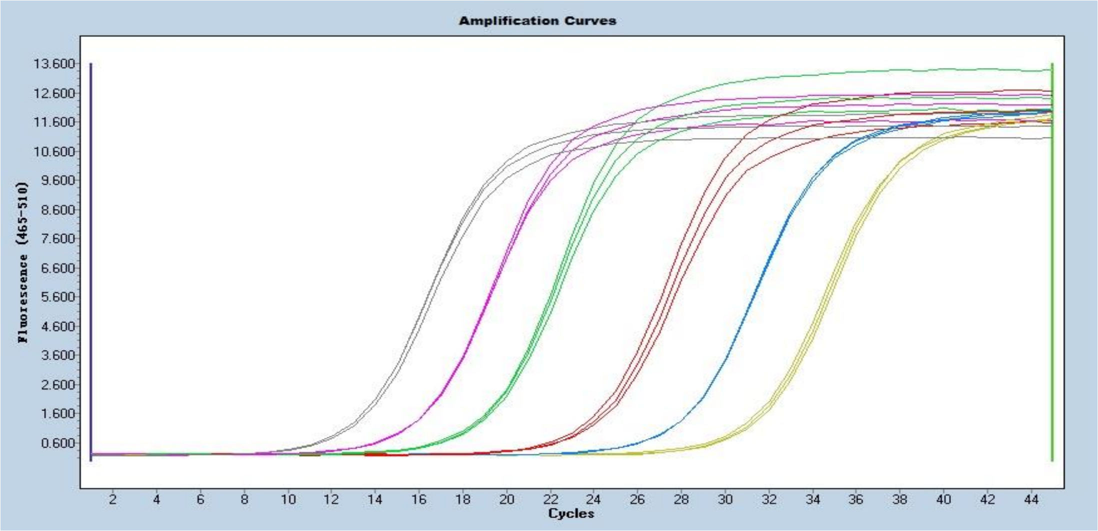

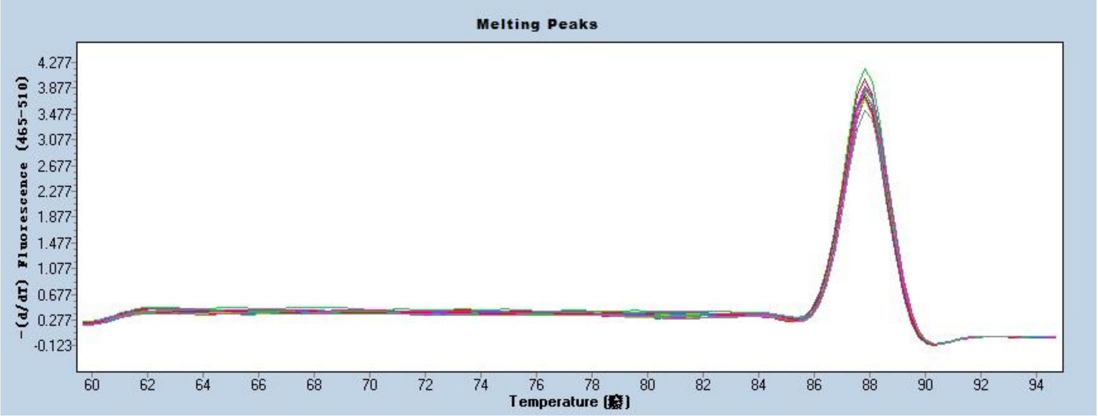

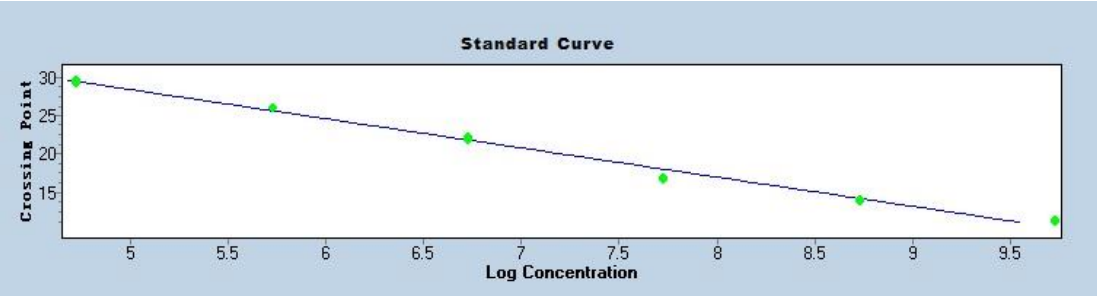

Note: Ordinate is CT；abscissa LogCO，LogCO is Log concentration (sample concentration of logarithm)

Slope=-3.777

Amplification efficiency: $E=10^{-1/slope}-1=10^{-1/-3.777}-1=84.0\%$

Correlation coefficient: $R^2=0.9901$

Y Intercept: 47.23

Error: 0.247

phtC:

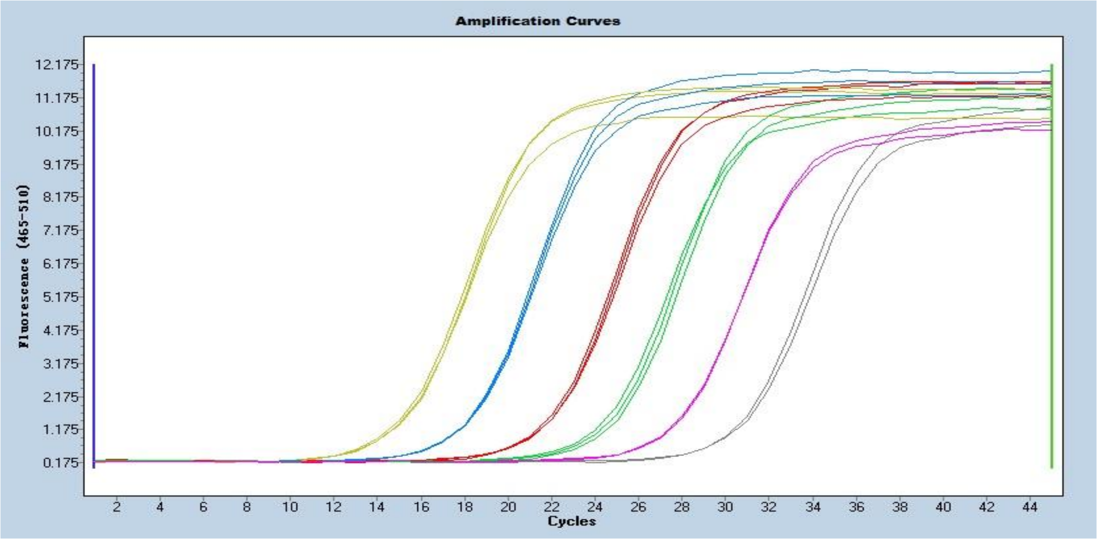

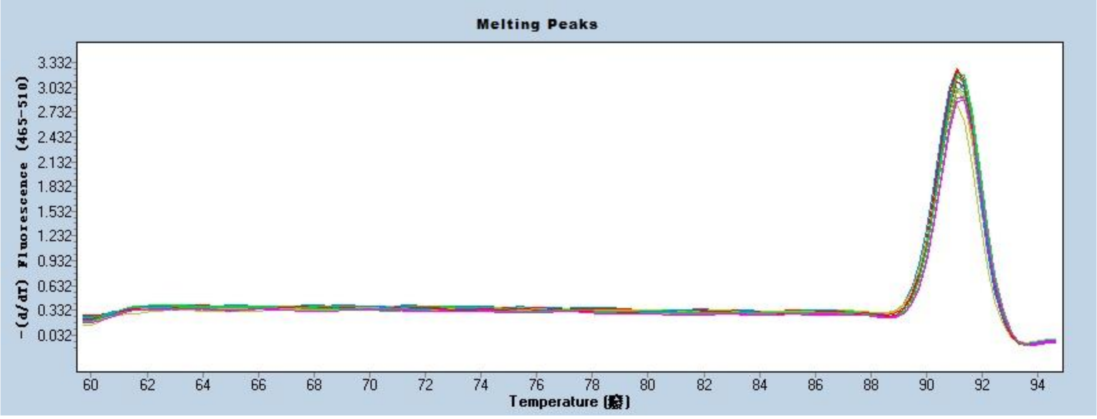

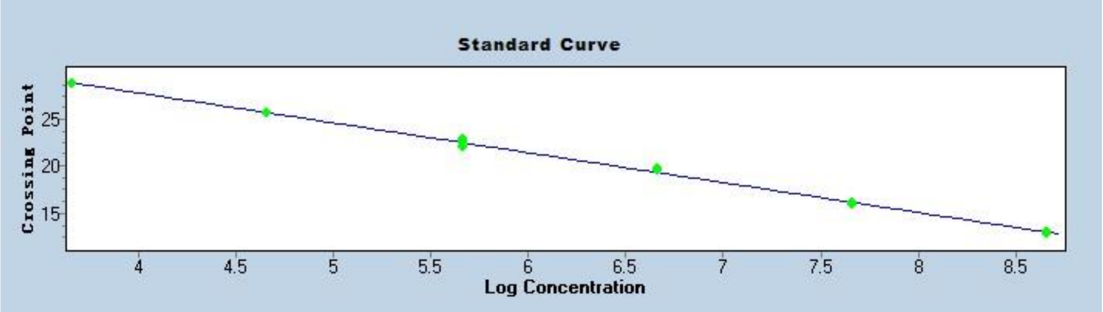

Note: Ordinate is CT；abscissa LogCO，LogCO is Log concentration (sample concentration of logarithm)

Slope=-3.157

Amplification efficiency: $E=10^{-1/slope}-1=10^{-1/-3.157}-1=107.4\%$

Correlation coefficient: $R^2=0.9983$

Y Intercept: 40.33

Error: 0.0606

Supplement Table 1. The active link of metabolic pathway

		Link
Nitrogen metabolism	CK	http://www.kegg.jp/kegg-bin/show_pathway?ko00910+K00260+K01673+K00370+K01745+K00265+K00266+K01915+K00362+K00262+K00368+K00372+K01953+K00605+K01744+K00371+K00459+K01668+K01425+K00284+K01455+K04561+K00926+K04748+K01916+K00261+K01424+K01667+K00366+K01501+K01760+K14155+K02594+K00285+K00376
	DBP1	http://www.kegg.jp/kegg-bin/show_pathway?ko00910+K00260+K01673+K00370+K01745+K00265+K00266+K01915+K00362+K00262+K00368+K00372+K01953+K00605+K01744+K00371+K00459+K01668+K01425+K00284+K01455+K04561+K00926+K04748+K01916+K00261+K00374+K01424+K01667+K00373+K00366+K01501+K01760+K14155+K02594+K00360+K00363+K00285
	DBP2	http://www.kegg.jp/kegg-bin/show_pathway?ko00910+K00260+K01673+K00370+K01745+K00265+K00266+K01915+K00362+K00262+K00368+K00372+K01953+K00605+K01744+K00371+K00459+K01668+K01425+K00284+K01455+K04561+K00926+K04748+K01916+K00261+K00374+K01424+K01667+K00373+K00366+K01501+K01760+K14155+K02594+K00363+K00285+K02164
	DBP3	http://www.kegg.jp/kegg-bin/show_pathway?ko00910+K00260+K01673+K00370+K01745+K00265+K00266+K01915+K00362+K00262+K00368+K00372+K01953+K00605+K01744+K00371+K00459+K01668+K01425+K00284+K01455+K04561+K00926+K04748+K01916+K00261+K00374+K01424+K01667+K00373+K00366+K01501+K01760+K14155+K02594+K00360+K00363+K00285+K02164
	DBP4	http://www.kegg.jp/kegg-bin/show_pathway?ko00910+K00260+K01673+K00370+K01745+K00265+K00266+K01915+K00362+K00262+K00368+K00372+K01953+K00605+K01744+K00371+K00459+K01668+K01425+K00284+K01455+K04561+K00926+K04748+K01916+K00261+K00374+K01424+K01667+K00373+K00366+K01501+K01760+K14155+K02594+K00360+K00363+K00285+K02164
Glycolysis / Gluconeogene	CK	http://www.kegg.jp/kegg-bin/show_pathway?ko00010+K01895+K01810+K00382+K01610+K01222+K00627+K00114+K00163+K00845+K01689+K03841+K00134+K00121+K00161+K00873+K13810+K00162+K13953+K00850+K00128+K02446+K00927+K01596+K00016+K00170+K01623+K01834+K01624+K00886+K01622+K04072+K00001+K01803+K04041+K01223+K00150+K01835+K01785+K00169+K00131+K00172+K02777+K00171
	D	http://www.kegg.jp/kegg-

sis	BP1	bin/show_pathway?ko00010+K01895+K01810+K00382+K01610+K01222+K00627+K00114+K00163+K00845+K01689+K03841+K00134+K00121+K00161+K00873+K13810+K00162+K13953+K00850+K00128+K02446+K00927+K01596+K00016+K00170+K01623+K01834+K01624+K00886+K01622+K04072+K00001+K01803+K04041+K01223+K00150+K01835+K01785+K00169+K00131+K00172+K02777+K00171
	DBP2	http://www.kegg.jp/kegg-bin/show_pathway?ko00010+K01895+K01810+K00382+K01610+K01222+K00627+K00114+K00163+K00845+K01689+K03841+K00134+K00121+K00161+K00873+K13810+K00162+K13953+K00850+K00128+K02446+K00927+K01596+K00016+K00170+K01623+K01834+K01624+K00886+K01622+K04072+K00001+K01803+K04041+K01223+K00150+K01835+K01785+K00169+K00131+K00172+K02777+K00171+K01792
	DBP3	http://www.kegg.jp/kegg-bin/show_pathway?ko00010+K01895+K01810+K00382+K01610+K01222+K00627+K00114+K00163+K00845+K01689+K03841+K00134+K00121+K00161+K00873+K13810+K00162+K13953+K00850+K00128+K02446+K00927+K01596+K00016+K00170+K01623+K01834+K01624+K00886+K01622+K04072+K00001+K01803+K04041+K01223+K00150+K01835+K01785+K00169+K00131+K00172+K02777+K00171+K01792
	DBP4	http://www.kegg.jp/kegg-bin/show_pathway?ko00010+K01895+K01810+K00382+K01610+K01222+K00627+K00114+K00163+K00845+K01689+K03841+K00134+K00121+K00161+K00873+K13810+K00162+K13953+K00850+K00128+K02446+K00927+K01596+K00016+K00170+K01623+K01834+K01624+K00886+K01622+K04072+K00001+K01803+K04041+K01223+K00150+K01835+K01785+K00169+K00131+K00172+K02777+K00171+K01792
Citrate cycle (TCA cycle)	CK	http://www.kegg.jp/kegg-bin/show_pathway?ko00020+K01681+K00174+K00239+K01958+K00382+K00658+K01610+K00627+K00163+K01647+K01679+K01903+K00164+K00161+K00162+K00030+K00175+K01596+K00170+K00031+K01676+K01902+K01677+K01644+K00240+K00024+K00244+K01678+K00169+K01960+K01959+K00245+K00172+K00242+K00241+K00247+K00171+K00246
	DBP1	http://www.kegg.jp/kegg-bin/show_pathway?ko00020+K01681+K00174+K00239+K01958+K00382+K00658+K01610+K00627+K00163+K01647+K01679+K01903+K00164+K00161+K00162+K00030+K00175+K01596+K00170+K00031+K01676+K01902+K01677+K01644+K00240+K00024+K01682+K00244+K01678+K00169+K01960+K01959+K00245+K00172+K00242+K00241+K00247+K00171+K00246
	D	http://www.kegg.jp/kegg-

	BP2	bin/show_pathway?ko00020+K01681+K00174+K00239+K01958+K00382+K00658+K01610+K00627+K00163+K01647+K01679+K01903+K00164+K00161+K00162+K00030+K00175+K01596+K00170+K00031+K01676+K01902+K01677+K01644+K00240+K00024+K01682+K00244+K01678+K00169+K01960+K01959+K00245+K00172+K00242+K00241+K00247+K00171+K00246
	DBP3	http://www.kegg.jp/kegg-bin/show_pathway?ko00020+K01681+K00174+K00239+K01958+K00382+K00658+K01610+K00627+K00163+K01647+K01679+K01903+K00164+K00161+K00162+K00030+K00175+K01596+K00170+K00031+K01676+K01902+K01677+K01644+K00240+K00024+K01682+K00244+K01678+K00169+K01960+K01959+K00245+K00172+K00242+K00241+K00247+K00171+K00246
	DBP4	http://www.kegg.jp/kegg-bin/show_pathway?ko00020+K01681+K00174+K00239+K01958+K00382+K00658+K01610+K00627+K00163+K01647+K01679+K01903+K00164+K00161+K00162+K00030+K00175+K01596+K00170+K00031+K01676+K01902+K01677+K01644+K00240+K00024+K00244+K01678+K00169+K01960+K01959+K00245+K00172+K00242+K00241+K00247+K00171+K00246
Pentose phosphate pathway	CK	http://www.kegg.jp/kegg-bin/show_pathway?ko00030+K01621+K00615+K01810+K00036+K01839+K03841+K00852+K13810+K00850+K00616+K02446+K00874+K00033+K00948+K01623+K01619+K01624+K00851+K01057+K04041+K01690+K01835+K07404+K01783+K01625+K00117+K01807+K01808
	DBP1	http://www.kegg.jp/kegg-bin/show_pathway?ko00030+K01621+K00615+K01810+K00036+K01839+K03841+K00852+K13810+K00850+K00616+K02446+K00874+K00033+K00948+K01623+K01619+K01624+K00851+K01057+K04041+K01690+K01835+K07404+K01783+K01625+K00117+K01807+K01808
	DBP2	http://www.kegg.jp/kegg-bin/show_pathway?ko00030+K01621+K00615+K01810+K00036+K01839+K03841+K00852+K13810+K00850+K00616+K02446+K00874+K00033+K00948+K01623+K01619+K01624+K00851+K01057+K04041+K01690+K01835+K07404+K01783+K01625+K00117+K01807+K01808+K01053
	DBP3	http://www.kegg.jp/kegg-bin/show_pathway?ko00030+K01621+K00615+K01810+K00036+K01839+K03841+K00852+K13810+K00850+K00616+K02446+K00874+K00033+K00948+K01623+K01619+K01624+K00851+K01057+K04041+K01690+K01835+K07404+K01783+K01625+K00117+K06152+K01807+K01808+K01053
	DB	http://www.kegg.jp/kegg-bin/show_pathway?ko00030+K01621+K00615+K01810+K00036+K01839+K0

	P4	3841+K00852+K13810+K00850+K00616+K02446+K00874+K00033+K00948+K01623+K01619+K01624+K00851+K01057+K04041+K01690+K01835+K07404+K01783+K01625+K00117+K06152+K01807+K01808+K01053
Sulfur metabolism	CK	http://www.kegg.jp/kegg-bin/show_pathway?ko00920+K00381+K01739+K00641+K01738+K00958+K12339+K00956+K00390+K00651+K00957+K00860+K00640+K01760+K14155+K13811+K00955
	DBP1	http://www.kegg.jp/kegg-bin/show_pathway?ko00920+K00381+K01739+K00641+K01738+K00958+K12339+K00956+K00390+K00651+K00957+K00860+K00640+K01760+K14155+K13811
	DBP2	http://www.kegg.jp/kegg-bin/show_pathway?ko00920+K00381+K01739+K00641+K01738+K00958+K12339+K00956+K00390+K00651+K00957+K00860+K00640+K01760+K14155+K13811
	DBP3	http://www.kegg.jp/kegg-bin/show_pathway?ko00920+K00381+K01739+K00641+K01738+K00958+K12339+K00956+K00390+K00651+K00957+K00860+K00640+K01760+K14155+K13811+K00955
	DBP4	http://www.kegg.jp/kegg-bin/show_pathway?ko00920+K00381+K01739+K00641+K01738+K00958+K12339+K00956+K00390+K00651+K00957+K00860+K00640+K01760+K14155+K13811

Supplement Table 2. The link of signal regulatory pathways

		Link
Apoptosis	CK	http://www.kegg.jp/kegg-bin/show_pathway?ko04210+K04739+K01173
	DBP1	http://www.kegg.jp/kegg-bin/show_pathway?ko04210+K04739+K01173
	DBP2	http://www.kegg.jp/kegg-bin/show_pathway?ko04210+K04739+K01173
	DBP3	http://www.kegg.jp/kegg-bin/show_pathway?ko04210+K04739+K01173
	DBP4	http://www.kegg.jp/kegg-bin/show_pathway?ko04210+K04739+K01173
Glutamatergic synapse	CK	http://www.kegg.jp/kegg-bin/show_pathway?ko04724+K01915+K01425
	DBP1	http://www.kegg.jp/kegg-bin/show_pathway?ko04724+K01915+K01425
	DBP2	http://www.kegg.jp/kegg-bin/show_pathway?ko04724+K01915+K01425
	DBP3	http://www.kegg.jp/kegg-bin/show_pathway?ko04724+K01915+K01425
	DBP4	http://www.kegg.jp/kegg-bin/show_pathway?ko04724+K01915+K01425
Peroxisome	CK	http://www.kegg.jp/kegg-bin/show_pathway?ko04146+K00232+K01897+K03781+K03426+K00031+K00869+K04564+K01640+K01796
	DBP1	http://www.kegg.jp/kegg-bin/show_pathway?ko04146+K00232+K01897+K03781+K00803+K03426+K00031+K00624+K00869+K04564+K01640+K01796
	DBP2	http://www.kegg.jp/kegg-bin/show_pathway?ko04146+K00232+K01897+K03781+K00803+K03426+K00031+K00624+K00869+K04564+K01640+K01796
	DBP3	http://www.kegg.jp/kegg-bin/show_pathway?ko04146+K00232+K01897+K03781+K00803+K03426+K00031+K00624+K00869+K04564+K01640+K01796
	DBP4	http://www.kegg.jp/kegg-bin/show_pathway?ko04146+K00232+K01897+K03781+K00803+K03426+K00031+K00869+K04564+K01640+K01796

Supplementary Table 3. Soil DNA kit protocol

Working order	Detail Operation Description
1.	Transfer 500 mg glass beads to a 15 mL centrifuge tube.
2.	Add 0.2-1.0 g soil sample to the glass beads.
3.	Add 1 mL SLX-Mlus Buffer. Vortex at maximum speed for 3-5 minutes to lyse samples.Note: For best result, a mixer mill, such as GenoGrinder 2010, Fastprep-24®, Mixer Mill MM 300®, should be used.
4.	Add 100 μL DS Buffer. Vortex to mix thoroughly.
5.	Incubate at 70°C for 10 min. Briefly vortex the tube once during the incubation.
6.	Centrifuge at 3,000 rpm for 3 minutes at room temperature.
7.	Transfer 800 μL the supernatant into a new 2 mL microcentrifuge tube
8.	Add 270 μL P2 Buffer. Vortex to mix thoroughly.
9.	Incubate on ice for 5 minutes.
10.	Centrifuge at ≥13,000 x g for 5 minutes at 4°C.
11.	Carefully transfer the supernatant to a new 2 mL microcentrifuge tube.
12.	Add 0.7 volumes isopropanol. Mix thoroughly by inverting tube for 20-30 times Note: If the soil contains very low DNA, incubate the sample at -20°C for 1 hour.
13.	Centrifuge at ≥13,000 x g for 10 minutes at 4°C
14.	Carefully aspirate and discard the supernatant. Do not disturb the DNA pellet.
15.	Invert the tube on a absorbent paper for 1 minute to drain the liquid.<br>Note: It is not necessary to dry the DNA pellet.
16.	Add 200 μL Elution Buffer. Vortex for 10 seconds.
17.	Incubate at 70°C for 10-20 minutes to dissolve the DNA pellet.
18.	Add 100 μL HTR Reagent. Vortex to mix thoroughly. Note: Completely resuspend HTR Reagent by shaking the bottle before use.
19.	Let sit at room temperature for 2 minutes.
20.	Centrifuge at ≥13,000 x g for 2 minutes.
21.	Transfer cleared supernatant to a new 2 mL microcentrifuge tube.<br>Note: If supernatant still has a dark color from the soil, repeat Steps 18-20 for a second HTR Reagent step.
22.	Add an equal volume XP1 Buffer. Vortex to mix thoroughly.
23.	Insert a HiBind® DNA Mini Column into a 2 mL Collection Tube provided in this kit.
24.	Transfer the sample from Step 22 to the HiBind® DNA Mini Column.
25.	Centrifuge at 10,000 x g for 1 minute at room temperature.
26.	Discard the filtrate and reuse the Collection Tube.
27.	Add 300 μL XP1 Buffer.
28.	Centrifuge at 10,000 x g for 1 minute.
29.	Discard the filtrate and the Collection Tube.
30.	Transfer the HiBind® DNA Mini Column into a new 2 mL Collection Tube.
31.	Add 700 μL SPW Wash Buffer. Note: SPW Wash Buffer must be diluted with ethanol before use. Please see the
32.	Centrifuge at 10,000 x g for 1 minute.

33.	Discard the filtrate and reuse the Collection Tube.
34.	Repeat Steps 31-33 for a second SPW Wash Buffer wash step.
35.	Centrifuge the empty HiBind® DNA Mini Column at ≥13,000 x g for 2 minutes at room temperature.<br>Note: This step is critical in removing residual ethanol that may interfere with downstream applications.
36.	Transfer the HiBind® DNA Mini Column into a clean 1.5 mL microcentrifuge tube.
37.	Add 30-100 µL Elution Buffer preheated to 70°C directly onto the center of HiBind®<br>membrane.
38.	Incubate at 70°C for 10-15 minutes.
39.	Centrifuge at ≥13,000 x g for 1 minute.
40.	Repeat Steps 37-39 for a second elution step.
41.	Discard the HiBind® DNA Mini Column and store eluted DNA at -20°C.

Supplementary Table 4. Setting parameters of M220 Focused-ultrasonicator™ (Covaris Inc., Woburn, MA, USA)

Setting	350 bp Insert	550 bp Insert
Duty factor	20%	
Peak Incident Power	50W	
Cycly per burst	200	
Duration	65 seconds	45seconds
Temperature	20℃	

Supplement Table 5. Statistics analysis on the metagenomics sequencing assembly and annotation

	CK	DBP1	DBP2	DBP3	DBP4
Raw reads	57,076,924	68,207,808	66,563,632	67,855,938	61,800,060
Clean reads	55,756,081	66,847,845	65,164,814	66,334,743	60,363,644
Percent in raw read (%)[a]	97.6859	98.0061	97.8985	97.7582	97.6757
Assembled contigs	4,891	8,219	6,435	12,520	11,701
Contig_N50 (bp)[b]	689	679	677	730	817
Contig_N90 (bp)[c]	525	523	523	530	532
Predicted ORFs	6,581	11,427	8,849	17,692	17,727
Total length (bp)	2,979,767	5,156,369	4,000,683	8,218,631	8,892,949

[a] It was the percentage of clean reads in raw reads

[b] Contig_N50 is a value that is determined when the length of the sequence was added up (from big to small) until over 50% of the total sequences for the first time.

[c] Contig_N90 is a value that is determined when the length of the sequence was added up (from big to small) until over 50% of the total sequences for the first time.

Supplementary Table 6. DNA concentration from the different soils

**Sample name**	**Conc. (ng/ul)**
CK	54.83±4.76
DBP1	42.11±5.23
DBP2	51.7±6.87
DBP3	48.32±5.98
DBP4	35.14±6.59

Supplementary Table 7-1. Analysis of variance for the total abundance genes of nitrogen cycling

Source	Degrees of freedom	Sum of squares	Mean square	*F* ratio	*P*
Between classes	$K - 1 = 4$	SS_B = 39134928.40	B = 9783732.10	B/W = 25.71	<0.001
Within classes (residual)	$N - K = 10$	SS_W= 3805101.33	$W = s^2_w$ = 380510.13		
Total	$N - 1 = 14$	SS_T = 42940029.73			

N is the total number of observations, i.e. units in the experiment or sampling sites in a survey.
K is the number of experimental treatments or strata in a survey.
SS_B, SS_W and SS_T are the sums of squares between treatments, within treatments and total, respectively.

Supplementary Table 7-2. Analysis of variance for the abundances of nitrate reductase (*NarGHIJ*, *NasAB* and *NxrAB* genes) gene

Source	Degrees of freedom	Sum of squares	Mean square	*F* ratio	*P*
Between classes	$K - 1 = 4$	SS_B = 521618.67	B = 130404.67	B/W = 136.06	<0.001
Within classes (residual)	$N - K = 10$	SS_W= 9584.67	$W = s^2_w$ = 958.47		
Total	$N - 1 = 14$	SS_T = 531203.33			

Supplementary Table 7-3. Analysis of variance for the abundances of nitrite reductase (*NirBD* gene) gene

Source	Degrees of freedom	Sum of squares	Mean square	*F* ratio	*P*
Between classes	$K - 1 = 4$	SS_B = 520926.40	B = 130231.60	B/W = 234.76	<0.001
Within classes (residual)	$N - K = 10$	SS_W= 5547.33	$W = s^2_w$		

			= 554.73		
Total	$N-1=14$	SS_T = 526473.73			

Supplementary Table 7-4. Analysis of variance for the abundances of formamidase gene

Source	Degrees of freedom	Sum of squares	Mean square	*F* ratio	*P*
Between classes	$K-1=4$	SS_B = 4305.60	*B* = 1076.40	*B*/*W* = 32.49	<0.001
Within classes (residual)	$N-K=10$	SS_W= 331.33	$W=s^2_w$ = 33.13		
Total	$N-1=14$	SS_T = 4636.93			

Supplementary Table 7-5. Analysis of variance for the abundances of nitrilase gene

Source	Degrees of freedom	Sum of squares	Mean square	*F* ratio	*P*
Between classes	$K-1=4$	SS_B = 846.40	*B* = 211.60	*B*/*W* = 7.64	<0.05
Within classes (residual)	$N-K=5$	SS_W= 138.50	$W=s^2_w$ = 27.70		
Total	$N-1=9$	SS_T = 984.90			

Supplementary Table 7-6. Analysis of variance for the abundances of nitronate monooxygenase gene

Source	Degrees of freedom	Sum of squares	Mean square	*F* ratio	*P*
Between classes	$K-1=4$	SS_B = 13593.07	*B* = 3398.27	*B*/*W* = 177.61	<0.001
Within classes (residual)	$N-K=5$	SS_W= 191.33	$W=s^2_w$ = 19.13		
Total	$N-1=9$	SS_T = 13784.40			

Supplementary Table 7-7. Analysis of variance for the abundances of nitric oxide

reductase (*NorBC* gene) gene

Source	Degrees of freedom	Sum of squares	Mean square	*F* ratio	*P*
Between classes	$K - 1 = 4$	SS_B = 1041.60	B = 260.4	B/W = 43.89	<0.001
Within classes (residual)	$N - K = 5$	SS_W= 59.33	$W = s^2_w$ = 5.93		
Total	$N - 1 = 9$	SS_T = 1100.93			

Supplementary Table 7-8. Analysis of variance for the abundances of ferredoxin-nitrite reductase (*NirA* gene) gene

Source	Degrees of freedom	Sum of squares	Mean square	*F* ratio	*P*
Between classes	$K - 1 = 4$	SS_B = 17484.27	B = 4371.07	B/W = 188.95	<0.001
Within classes (residual)	$N - K = 5$	SS_W= 231.33	$W = s^2_w$ = 23.13		
Total	$N - 1 = 9$	SS_T = 17715.60			

Supplementary Table 8-1. Analysis of variance for the total abundance genes of glycolysis

Source	Degrees of freedom	Sum of squares	Mean square	F ratio	P
Between classes	$K - 1 = 4$	SS_B = 74099838.67	B = 18524959.67	B/W = 27.15	<0.001
Within classes (residual)	$N - K = 10$	SS_W= 6822532.67	$W = s^2_w$ = 682253.27		
Total	$N - 1 = 14$	SS_T = 80922371.33			

N is the total number of observations, i.e. units in the experiment or sampling sites in a survey.
K is the number of experimental treatments or strata in a survey.
SS_B, SS_W and SS_T are the sums of squares between treatments, within treatments and total, respectively.

Supplementary Table 8-2. Analysis of variance for the abundances of pyruvate kinase gene in glycolysis

Source	Degrees of freedom	Sum of squares	Mean square	F ratio	P
Between classes	$K - 1 = 4$	SS_B = 72050.67	B = 18012.67	B/W = 109.34	<0.001
Within classes (residual)	$N - K = 10$	SS_W= 1647.33	$W = s^2_w$ = 164.73		
Total	$N - 1 = 14$	SS_T = 73698			

Supplementary Table 8-3. Analysis of variance for the abundances of glucokinase gene in glycolysis

Source	Degrees of freedom	Sum of squares	Mean square	F ratio	P
Between classes	$K - 1 = 4$	SS_B = 66336.60	B = 16584.15	B/W = 11.83	<0.01
Within classes (residual)	$N - K = 5$	SS_W= 7007.00	$W = s^2_w$ = 1401.40		

Total	$N - 1 = 9$	SS_T = 73343.60			

Supplementary Table 8-4. Analysis of variance for the abundances of 6-phosphofructokinase gene in glycolysis

Source	Degrees of freedom	Sum of squares	Mean square	*F* ratio	*P*
Between classes	$K - 1 = 4$	SS_B = 62092.40	B = 15523.10	B/W = 17.16	<0.001
Within classes (residual)	$N - K = 5$	SS_W= 9045.33	$W = s^2_w$ = 904.53		
Total	$N - 1 = 9$	SS_T = 71137.73			

Supplementary Table 8-5. Analysis of variance for the abundances of phosphoglucomutase gene in glycolysis

Source	Degrees of freedom	Sum of squares	Mean square	*F* ratio	*P*
Between classes	$K - 1 = 4$	SS_B = 3902.40	B = 975.60	B/W = 136.77	<0.001
Within classes (residual)	$N - K = 10$	SS_W= 71.33	$W = s^2_w$ = 7.13		
Total	$N - 1 = 14$	SS_T = 3973.73			

Supplementary Table 8-6. Analysis of variance for the abundances of L-lactate dehydrogenase gene in glycolysis

Source	Degrees of freedom	Sum of squares	Mean square	*F* ratio	*P*
Between classes	$K - 1 = 4$	SS_B = 13457.60	B = 3364.40	B/W = 71.74	<0.001
Within classes (residual)	$N - K = 5$	SS_W= 234.50	$W = s^2_w$ = 46.9		
Total	$N - 1 = 9$	SS_T = 13692.10			

Supplementary Table 8-7. Analysis of variance for the abundances of polyphosphate-glucose phosphotransferase gene in glycolysis

Source	Degrees of freedom	Sum of squares	Mean square	F ratio	P
Between classes	$K - 1 = 4$	SS_B = 9926.40	B = 2481.60	B/W = 85.18	<0.001
Within classes (residual)	$N - K = 10$	SS_W= 291.33	$W = s^2_w$ = 29.13		
Total	$N - 1 = 14$	SS_T = 10217.73			

Supplementary Table 8-8. Analysis of variance for the abundances of phosphoenolpyruvate carboxykinase gene in glycolysis

Source	Degrees of freedom	Sum of squares	Mean square	F ratio	P
Between classes	$K - 1 = 4$	SS_B = 188601.60	B = 47150.40	B/W = 242.13	<0.001
Within classes (residual)	$N - K = 10$	SS_W= 1947.33	$W = s^2_w$ = 194.73		
Total	$N - 1 = 14$	SS_T = 190548.93			

Supplementary Table 8-9. Analysis of variance for the abundances of pyruvate dehydrogenase gene in glycolysis

Source	Degrees of freedom	Sum of squares	Mean square	F ratio	P
Between classes	$K - 1 = 4$	SS_B = 2178961.73	B = 544740.43	B/W = 34.25	<0.001
Within classes (residual)	$N - K = 10$	SS_W= 159028.67	$W = s^2_w$ = 15902.87		
Total	$N - 1 = 14$	SS_T = 2337990.40			

Supplementary Table 8-10. Analysis of variance for the abundances of acetate-CoA

ligase gene in glycolysis

Source	Degrees of freedom	Sum of squares	Mean square	F ratio	P
Between classes	$K - 1 = 4$	SS_B = 1421924.27	B = 355481.07	B/W = 49.49	<0.001
Within classes (residual)	$N - K = 10$	SS_W= 71825.33	$W = s^2_w$ = 7182.53		
Total	$N - 1 = 14$	SS_T = 1493749.60			

Supplementary Table 8-11. Analysis of variance for the abundances of alcohol dehydrogenase gene in glycolysis

Source	Degrees of freedom	Sum of squares	Mean square	F ratio	P
Between classes	$K - 1 = 4$	SS_B = 157002.00	B = 39250.50	B/W = 13.41	<0.001
Within classes (residual)	$N - K = 10$	SS_W= 29277.33	$W = s^2_w$ = 2927.73		
Total	$N - 1 = 14$	SS_T = 186279.33			

Supplementary Table 8-12. Analysis of variance for the abundances of aldehyde dehydrogenase gene in glycolysis

Source	Degrees of freedom	Sum of squares	Mean square	F ratio	P
Between classes	$K - 1 = 4$	SS_B = 312965.07	B = 78241.27	B/W = 29.50	<0.001
Within classes (residual)	$N - K = 10$	SS_W= 26523.33	$W = s^2_w$ = 2652.33		
Total	$N - 1 = 14$	SS_T = 339488.40			

Supplementary Table 8-13. Analysis of variance for the abundances of dihydrolipoyl dehydrogenase gene in glycolysis

Source	Degrees of freedom	Sum of squares	Mean square	F ratio	P
Between classes	$K-1=4$	SS_B = 360246.27	B = 90061.57	B/W = 46.40	<0.001
Within classes (residual)	$N-K=10$	SS_W= 19411.33	$W=s^2_w$ = 1941.13		
Total	$N-1=14$	SS_T = 379657.60			

Supplementary Table 8-14. Analysis of variance for the abundances of phosphoglycerate mutase gene in glycolysis

Source	Degrees of freedom	Sum of squares	Mean square	F ratio	P
Between classes	$K-1=4$	SS_B = 129072.27	B = 32268.07	B/W = 12.50	<0.001
Within classes (residual)	$N-K=10$	SS_W= 25824.67	$W=s^2_w$ = 2582.47		
Total	$N-1=14$	SS_T = 154896.93			

Supplementary Table 8-15. Analysis of variance for the abundances of dihydrolipoyllysine-residue acetyltransferase gene in glycolysis

Source	Degrees of freedom	Sum of squares	Mean square	F ratio	P
Between classes	$K-1=4$	SS_B = 467902.00	B = 116975.50	B/W = 94.05	<0.001
Within classes (residual)	$N-K=10$	SS_W= 12437.33	$W=s^2_w$ = 1243.73		
Total	$N-1=14$	SS_T = 480339.33			

Supplementary Table 9-1. Analysis of variance for the total abundance genes of TCA cycle

Source	Degrees of freedom	Sum of squares	Mean square	F ratio	P
Between classes	$K-1=4$	SS_B = 54813974.40	B = 13703493.60	B/W = 21.68	<0.001
Within classes (residual)	$N-K=10$	SS_W= 6321099.33	$W=s^2_w$ = 632109.93		
Total	$N-1=14$	SS_T = 61135073.73			

N is the total number of observations, i.e. units in the experiment or sampling sites in a survey.
K is the number of experimental treatments or strata in a survey.
SS_B, SS_W and SS_T are the sums of squares between treatments, within treatments and total, respectively.

Supplementary Table 9-2. Analysis of variance for the abundances of Oxoglutarate dehydrogenase gene in TCA cycle

Source	Degrees of freedom	Sum of squares	Mean square	F ratio	P
Between classes	$K-1=4$	SS_B = 165591.60	B = 41397.90	B/W = 41.37	<0.001
Within classes (residual)	$N-K=10$	SS_W= 10007.33	$W=s^2_w$ = 1000.73		
Total	$N-1=14$	SS_T = 175598.93			

Supplementary Table 9-3. Analysis of variance for the abundances of isocitrate dehydrogenase gene in TCA cycle

Source	Degrees of freedom	Sum of squares	Mean square	F ratio	P
Between classes	$K-1=4$	SS_B = 228321.73	B = 57080.43	B/W = 54.68	<0.001
Within classes (residual)	$N-K=10$	SS_W= 10440.00	$W=s^2_w$		

			= 1044.00		
Total	$N - 1 = 14$	SS_T = 238761.73			

Supplementary Table 9-4. Analysis of variance for the abundances of citrate (Si)-synthase gene in TCA cycle

Source	Degrees of freedom	Sum of squares	Mean square	*F* ratio	*P*
Between classes	$K - 1 = 4$	SS_B = 120266.00	B = 30066.50	B/W = 21.59	<0.001
Within classes (residual)	$N - K = 10$	SS_W= 13929.33	$W = s^2_w$ = 1392.93		
Total	$N - 1 = 14$	SS_T = 134195.33			

Supplementary Table 9-5. Analysis of variance for the abundances of pyruvate carboxylase gene in TCA cycle

Source	Degrees of freedom	Sum of squares	Mean square	*F* ratio	*P*
Between classes	$K - 1 = 4$	SS_B = 167970.40	B = 41992.60	B/W = 187.36	<0.001
Within classes (residual)	$N - K = 10$	SS_W= 2241.33	$W = s^2_w$ = 224.13		
Total	$N - 1 = 14$	SS_T = 170211.73			

Supplementary Table 9-6. Analysis of variance for the abundances of dihydrolipoyllysine-residue succinyltransferase gene in TCA cycle

Source	Degrees of freedom	Sum of squares	Mean square	*F* ratio	*P*
Between classes	$K - 1 = 4$	SS_B = 55752.40	B = 13938.10	B/W = 33.32	<0.001
Within classes (residual)	$N - K = 10$	SS_W= 4183.33	$W = s^2_w$		

			= 418.33		
Total	$N - 1 = 14$	SS_T = 59935.73			

Supplementary Table 9-7. Analysis of variance for the abundances of fumarate hydratase gene in TCA cycle

Source	Degrees of freedom	Sum of squares	Mean square	*F* ratio	*P*
Between classes	$K - 1 = 4$	SS_B = 79420.60	B = 19855.15	$B/W = 8.34$	<0.05
Within classes (residual)	$N - K = 5$	SS_W= 11899.50	$W = s^2_w$ = 2379.90		
Total	$N - 1 = 9$	SS_T = 91320.10			

Supplementary Table 9-8. Analysis of variance for the abundances of succinate-CoA ligase gene in TCA cycle

Source	Degrees of freedom	Sum of squares	Mean square	*F* ratio	*P*
Between classes	$K - 1 = 4$	SS_B = 99636.40	B = 24909.10	$B/W = 9.85$	<0.01
Within classes (residual)	$N - K = 10$	SS_W= 25285.33	$W = s^2_w$ = 2528.53		
Total	$N - 1 = 14$	SS_T = 124921.73			

Supplementary Table 9-9. Analysis of variance for the abundances of aconitate hydratase gene in TCA cycle

Source	Degrees of freedom	Sum of squares	Mean square	*F* ratio	*P*
Between classes	$K - 1 = 4$	SS_B = 294280.40	B = 73570.10	B/W = 18.10	<0.001
Within classes (residual)	$N - K = 10$	SS_W= 40649.33	$W = s^2_w$ = 4064.93		

Total	$N – 1 = 14$	SS_T = 334929.73			

Supplementary Table 9-10. Analysis of variance for the abundances of oxoglutarate synthase gene in TCA cycle

Source	Degrees of freedom	Sum of squares	Mean square	*F* ratio	*P*
Between classes	$K – 1 = 4$	SS_B = 266842.00	B = 66710.50	B/W = 11.89	<0.001
Within classes (residual)	$N – K = 10$	SS_W= 56129.33	$W = s^2_w$ = 5612.93		
Total	$N – 1 = 14$	SS_T = 322971.33			

Supplementary Table 10-1. Analysis of variance for the total abundance genes of pentose phosphate pathways

Source	Degrees of freedom	Sum of squares	Mean square	*F* ratio	*P*
Between classes	$K - 1 = 4$	SS_B = 22624703.60	*B* = 5656175.90	*B*/*W* = 25.65	<0.001
Within classes (residual)	$N - K = 10$	SS_W= 2204949.33	$W = s^2_w$ = 220494.93		
Total	$N - 1 = 14$	SS_T = 24829652.93			

N is the total number of observations, i.e. units in the experiment or sampling sites in a survey.
K is the number of experimental treatments or strata in a survey.
SS_B, SS_W and SS_T are the sums of squares between treatments, within treatments and total, respectively.

Supplementary Table 10-2. Analysis of variance for the abundances of phosphogluconate dehydrogenase gene in pentose phosphate pathways

Source	Degrees of freedom	Sum of squares	Mean square	*F* ratio	*P*
Between classes	$K - 1 = 4$	SS_B = 172059.33	*B* = 43014.83	*B*/*W* = 68.29	<0.001
Within classes (residual)	$N - K = 10$	SS_W= 6298.67	$W = s^2_w$ = 629.87		
Total	$N - 1 = 14$	SS_T = 178358.00			

Supplementary Table 10-3. Analysis of variance for the abundances of glucose-6-phosphate dehydrogenase gene in pentose phosphate pathways

Source	Degrees of freedom	Sum of squares	Mean square	*F* ratio	*P*
Between classes	$K - 1 = 4$	SS_B = 197358.00	*B* = 49339.50	*B*/*W* = 21.90	<0.001
Within classes (residual)	$N - K = 10$	SS_W= 22525.33	$W = s^2_w$		

			= 2252.53		
Total	$N - 1 = 14$	SS_T = 219883.33			

Supplementary Table 10-4. Analysis of variance for the abundances of phosphoglucomutase gene in pentose phosphate pathways

Source	Degrees of freedom	Sum of squares	Mean square	*F* ratio	*P*
Between classes	$K - 1 = 4$	SS_B = 3902.40	B = 975.60	B/W = 190.05	<0.001
Within classes (residual)	$N - K = 10$	SS_W= 51.33	$W = s^2_w$ = 5.13		
Total	$N - 1 = 14$	SS_T = 3953.73			

Supplementary Table 10-5. Analysis of variance for the abundances of 2-dehydro-3-deoxygluconokinase gene in pentose phosphate pathways

Source	Degrees of freedom	Sum of squares	Mean square	*F* ratio	*P*
Between classes	$K - 1 = 4$	SS_B = 19462.67	B = 4865.67	B/W = 97.44	<0.001
Within classes (residual)	$N - K = 10$	SS_W= 499.33	$W = s^2_w$ = 49.93		
Total	$N - 1 = 14$	SS_T = 19962.00			

Supplementary Table 10-6. Analysis of variance for the abundances of 6-phosphogluconolactonase gene in pentose phosphate pathways

Source	Degrees of freedom	Sum of squares	Mean square	*F* ratio	*P*
Between classes	$K - 1 = 4$	SS_B = 17612.27	B = 4403.07	B/W = 25.85	<0.001
Within classes (residual)	$N - K = 10$	SS_W= 1703.33	$W = s^2_w$ = 170.33		

Total	$N - 1 = 14$	SS_T = 19315.60			

Supplementary Table 10-7. Analysis of variance for the abundances of 6-phosphofructokinase gene in pentose phosphate pathways

Source	Degrees of freedom	Sum of squares	Mean square	*F* ratio	*P*
Between classes	$K - 1 = 4$	SS_B = 61993.07	B = 15498.27	B/W = 19.09	<0.001
Within classes (residual)	$N - K = 10$	SS_W= 8119.33	$W = s^2_w$ = 811.93		
Total	$N - 1 = 14$	SS_T = 70112.40			

Supplementary Table 10-8. Analysis of variance for the abundances of glucose-6-phosphate isomerase gene in pentose phosphate pathways

Source	Degrees of freedom	Sum of squares	Mean square	*F* ratio	*P*
Between classes	$K - 1 = 4$	SS_B = 84854.40	B = 21213.60	B/W = 15.84	<0.001
Within classes (residual)	$N - K = 10$	SS_W= 13391.33	$W = s^2_w$ = 1339.13		
Total	$N - 1 = 14$	SS_T = 98245.73			

Supplementary Table 10-9. Analysis of variance for the abundances of ribose-phosphate diphosphokinase gene in pentose phosphate pathways

Source	Degrees of freedom	Sum of squares	Mean square	*F* ratio	*P*
Between classes	$K - 1 = 4$	SS_B = 90304.00	B = 22576.00	B/W = 16.43	<0.001
Within classes (residual)	$N - K = 10$	SS_W= 13743.33	$W = s^2_w$ = 1374.33		

Total	$N - 1 = 14$	SS_T = 104047.33			

Supplementary Table 10-10. Analysis of variance for the abundances of transketolase gene in pentose phosphate pathways

Source	Degrees of freedom	Sum of squares	Mean square	F ratio	P
Between classes	$K - 1 = 4$	SS_B = 568108.27	B = 142027.07	B/W = 24.27	<0.001
Within classes (residual)	$N - K = 10$	SS_W= 58515.33	$W = s^2_w$ = 5851.53		
Total	$N - 1 = 14$	SS_T = 626623.60			

Supplementary Table 11-1. Analysis of variance for the total abundance genes of sulfur metabolism

Source	Degrees of freedom	Sum of squares	Mean square	*F* ratio	*P*
Between classes	$K - 1 = 4$	SS_B = 2132668.27	B = 533167.07	B/W = 21.69	<0.001
Within classes (residual)	$N - K = 10$	SS_W= 245811.33	$W = s^2_w$ = 24581.13		
Total	$N - 1 = 14$	SS_T = 2378479.60			

N is the total number of observations, i.e. units in the experiment or sampling sites in a survey.
K is the number of experimental treatments or strata in a survey.
SS_B, SS_W and SS_T are the sums of squares between treatments, within treatments and total, respectively.

Supplementary Table 11-2. Analysis of variance for the abundances of sulfate adenylyl-transferase gene in sulfur metabolism

Source	Degrees of freedom	Sum of squares	Mean square	*F* ratio	*P*
Between classes	$K - 1 = 4$	SS_B = 100569.73	B = 25142.43	B/W = 22.91	<0.001
Within classes (residual)	$N - K = 10$	SS_W= 10972.67	$W = s^2_w$ = 1097.27		
Total	$N - 1 = 14$	SS_T = 111542.40			

Supplementary Table 11-3. Analysis of variance for the abundances of sulfate reductase gene in sulfur metabolism

Source	Degrees of freedom	Sum of squares	Mean square	*F* ratio	*P*
Between classes	$K - 1 = 4$	SS_B = 6985.07	B = 1746.27	B/W = 3.83	<0.05
Within classes (residual)	$N - K = 10$	SS_W= 4559.33	$W = s^2_w$ = 455.93		

Total	$N - 1 = 14$	SS_T = 11544.40			

Supplementary Table 11-4. Analysis of variance for the abundances of sulfite reductase gene in sulfur metabolism

Source	Degrees of freedom	Sum of squares	Mean square	*F* ratio	*P*
Between classes	$K - 1 = 4$	SS_B = 17553.60	B = 4388.40	B/W = 18.78	<0.001
Within classes (residual)	$N - K = 10$	SS_W= 2337.33	$W = s^2_w$ = 233.73		
Total	$N - 1 = 14$	SS_T = 19890.93			

Supplementary Table 12-1. Analysis of variance for the total abundance genes of ABC transporters

Source	Degrees of freedom	Sum of squares	Mean square	F ratio	P
Between classes	$K-1=4$	SS_B = 337680748.40	B = 84420187.10	B/W = 27.45	<0.001
Within classes (residual)	$N-K=10$	SS_W= 30754595.33	$W=s^2_w$ = 3075459.53		
Total	$N-1=14$	SS_T = 368435343.70			

N is the total number of observations, i.e. units in the experiment or sampling sites in a survey.
K is the number of experimental treatments or strata in a survey.
SS_B, SS_W and SS_T are the sums of squares between treatments, within treatments and total, respectively.

Supplementary Table 12-2. Analysis of variance for the total abundance genes of two-component system (TCS)

Source	Degrees of freedom	Sum of squares	Mean square	F ratio	P
Between classes	$K-1=4$	SS_B = 67998310.27	B = 16999577.57	B/W = 25.81	<0.001
Within classes (residual)	$N-K=10$	SS_W= 6586894.67	$W=s^2_w$ = 658689.47		
Total	$N-1=14$	SS_T = 74585204.93			

Supplementary Table 12-3. Analysis of variance for the total abundance genes of phosphotransferase system (PTS)

Source	Degrees of freedom	Sum of squares	Mean square	F ratio	P
Between classes	$K-1=4$	SS_B = 2203134.00	B = 550783.50	B/W = 44.73	<0.001

Within classes (residual)	$N - K = 10$	SS_W= 123127.33	$W = s^2_w$ = 12312.73		
Total	$N - 1 = 14$	SS_T = 2326261.33			

Supplementary Table 12-4. Analysis of variance for the abundances of phosphotransferase gene in PTS

Source	Degrees of freedom	Sum of squares	Mean square	*F* ratio	*P*
Between classes	$K - 1 = 4$	SS_B = 19128.00	B = 4782.00	B/W = 116.26	<0.001
Within classes (residual)	$N - K = 10$	SS_W= 411.33	$W = s^2_w$ = 41.13		
Total	$N - 1 = 14$	SS_T = 19539.33			

Supplementary Table 12-5. Analysis of variance for the abundances of citryl-CoA lyase gene in TCS

Source	Degrees of freedom	Sum of squares	Mean square	*F* ratio	*P*
Between classes	$K - 1 = 4$	SS_B = 74784.40	B = 18696.10	B/W = 75.29	<0.001
Within classes (residual)	$N - K = 10$	SS_W= 2483.33	$W = s^2_w$ = 248.33		
Total	$N - 1 = 14$	SS_T = 77267.73			

Supplementary Table 12-6. Analysis of variance for the abundances of *Crr* and *BglF* gene in PTS

Source	Degrees of freedom	Sum of squares	Mean square	*F* ratio	*P*
Between classes	$K - 1 = 4$	SS_B = 48413.60	B = 12103.40	B/W = 67.57	<0.001

Within classes (residual)	$N - K = 10$	SS_W= 1791.33	$W = s^2_w$ = 179.13		
Total	$N - 1 = 14$	SS_T = 50204.93			

Supplementary Table 12-7. Analysis of variance for the abundances of monosaccharide-transporting ATPase gene in ABC transporters

Source	Degrees of freedom	Sum of squares	Mean square	*F* ratio	*P*
Between classes	$K - 1 = 4$	SS_B = 315573.60	B = 78893.40	B/W = 31.22	<0.001
Within classes (residual)	$N - K = 10$	SS_W= 25271.33	$W = s^2_w$ = 2527.13		
Total	$N - 1 = 14$	SS_T = 340844.93			

Supplementary Table 12-8. Analysis of variance for the abundances of malate dehydrogenase gene in TCS

Source	Degrees of freedom	Sum of squares	Mean square	*F* ratio	*P*
Between classes	$K - 1 = 4$	SS_B = 190674.67	B = 47668.67	B/W = 23.12	<0.001
Within classes (residual)	$N - K = 10$	SS_W= 20619.33	$W = s^2_w$ = 2061.93		
Total	$N - 1 = 14$	SS_T = 211294.00			

Supplementary Table 12-9. Analysis of variance for the abundances of *MalK* and *MsmK* gene in ABC transporters

Source	Degrees of freedom	Sum of squares	Mean square	*F* ratio	*P*
Between classes	$K - 1 = 4$	SS_B = 1520993.07	B = 380248.27	B/W = 17.95	<0.001

Within classes (residual)	$N - K = 10$	SS_W= 211787.33	$W = s^2_w$ = 21178.73		
Total	$N - 1 = 14$	SS_T = 1732780.40			

Supplementary Table 12-10. Analysis of variance for the abundances of histidine kinase gene in TCS

Source	Degrees of freedom	Sum of squares	Mean square	*F* ratio	*P*
Between classes	$K - 1 = 4$	SS_B = 1762603.73	*B* = 440650.93	*B*/*W* = 18.06	<0.001
Within classes (residual)	$N - K = 10$	SS_W= 243970.67	$W = s^2_w$ = 24397.07		
Total	$N - 1 = 14$	SS_T = 2006574.40			
